# Exploring various polygenic risk scores for skin cancer in the phenomes of the Michigan genomics initiative and the UK Biobank with a visual catalog: *PRSWeb*

**DOI:** 10.1371/journal.pgen.1008202

**Published:** 2019-06-13

**Authors:** Lars G. Fritsche, Lauren J. Beesley, Peter VandeHaar, Robert B. Peng, Maxwell Salvatore, Matthew Zawistowski, Sarah A. Gagliano Taliun, Sayantan Das, Jonathon LeFaive, Erin O. Kaleba, Thomas T. Klumpner, Stephanie E. Moser, Victoria M. Blanc, Chad M. Brummett, Sachin Kheterpal, Gonçalo R. Abecasis, Stephen B. Gruber, Bhramar Mukherjee

**Affiliations:** 1 Department of Biostatistics, University of Michigan School of Public Health, Ann Arbor, Michigan, United States of America; 2 Center for Statistical Genetics, University of Michigan School of Public Health, Ann Arbor, Michigan, United States of America; 3 Division of Pain Medicine, Department of Anesthesiology, University of Michigan Medical School, Ann Arbor, Michigan, United States of America; 4 Institute for Healthcare Policy and Innovation, University of Michigan, Ann Arbor, Michigan, United States of America; 5 Central Biorepository, University of Michigan Medical School, Ann Arbor, Michigan, United States of America; 6 USC Norris Comprehensive Cancer Center, University of Southern California, Los Angeles, California, United States of America; 7 Michigan Institute for Data Science, University of Michigan, Ann Arbor, Michigan, United States of America; 8 Department of Epidemiology, University of Michigan School of Public Health, Ann Arbor, Michigan, United States of America; 9 University of Michigan Rogel Cancer Center, University of Michigan, Ann Arbor, Michigan, United States of America; Harvard School of Public Health, UNITED STATES

## Abstract

Polygenic risk scores (PRS) are designed to serve as single summary measures that are easy to construct, condensing information from a large number of genetic variants associated with a disease. They have been used for stratification and prediction of disease risk. The primary focus of this paper is to demonstrate how we can combine PRS and electronic health records data to better understand the shared and unique genetic architecture and etiology of disease subtypes that may be both related and heterogeneous. PRS construction strategies often depend on the purpose of the study, the available data/summary estimates, and the underlying genetic architecture of a disease. We consider several choices for constructing a PRS using data obtained from various publicly-available sources including the UK Biobank and evaluate their abilities to predict not just the primary phenotype but also secondary phenotypes derived from electronic health records (EHR). This study was conducted using data from 30,702 unrelated, genotyped patients of recent European descent from the Michigan Genomics Initiative (MGI), a longitudinal biorepository effort within Michigan Medicine. We examine the three most common skin cancer subtypes in the USA: basal cell carcinoma, cutaneous squamous cell carcinoma, and melanoma. Using these PRS for various skin cancer subtypes, we conduct a phenome-wide association study (PheWAS) within the MGI data to evaluate PRS associations with secondary traits. PheWAS results are then replicated using population-based UK Biobank data and compared across various PRS construction methods. We develop an accompanying visual catalog called *PRSweb* that provides detailed PheWAS results and allows users to directly compare different PRS construction methods.

## Author summary

In the study of genetically complex diseases, polygenic risk scores (PRS) synthesize information from multiple genetic risk factors to provide insight into a patient’s inherited risk of developing a disease based on his/her genetic profile. These risk scores can be explored in conjunction with health and disease information available in electronic medical records. PRS may be associated with diseases that may be related to or precursors of the underlying disease of interest. In this paper, we demonstrate how PRS can be used in concert with the medical phenome to better understand the etiology of disease subtypes nested within a broad disease classification. This is done by examining the shared and distinct genetic risk factors across the related but heterogeneous disease subtypes and also through our comparison of the secondary associations across the phenome corresponding to the subtype specific PRS. We consider several PRS construction methods in our study. This framework of analysis is enabled by access to electronic health records and genetics data. Leveraging and harnessing the rich data resources of the Michigan Genomics Initiative, a biorepository effort at Michigan Medicine, and the large population-based UK Biobank study, we investigated the primary and secondary disease associations with PRS constructed for the three most common types of skin cancer: melanoma, basal cell carcinoma and cutaneous squamous cell carcinoma.

## Introduction

The underlying risk factors of genetically complex diseases are numerous. Genome-wide association studies (GWAS) on thousands of diseases and traits have made great strides in uncovering a vast array of genetic variants that contribute to genetic predispositions to disease [1]. In order to harness the information from a large number of genetic variants, a popular approach is to summarize their contribution through polygenic risk scores (PRS). While the performance of PRS to predict disease outcomes at a population level has been modest for many diseases, including most cancers, PRS have successfully been applied for risk stratification of cohorts [2, 3] and recently have been used to screen a multitude of clinical phenotypes (collectively called the medical phenome) for secondary trait associations [4, 5]. The goal of these phenome-wide screenings is to uncover phenotypes that share genetic components with the primary trait that, if pre-symptomatic, could shed biological insights into the disease pathway and inform early interventions or screening efforts for individuals at risk. Phenome-wide studies using PRS rely on an easily-calculated single biomarker that combines information across a spectrum of genetic variants. Additionally, PRS may be routinely available in patients’ electronic health records (EHR) in the near future, making analyses based on PRS a useful route for agnostic interrogation of the medical phenome. Existing literature has explored how to construct PRS with respect to a single disease phenotype [6, 7]. In this paper, we demonstrate how polygenic risk scores can be used in concert with the medical phenome to better understand the etiology of disease subtypes nested within a broad disease classification. This is done by examining the shared and distinct genetic risk factors across the related but heterogeneous disease subtypes and also through our comparison of the secondary associations across the phenome corresponding to subtype specific PRS.

In the post-GWAS era and with the availability of large biobank data from multiple sources, there is great interest in using gene-based biomarkers such as PRS for risk stratification and exploration of disease etiology. However, it is not always clear how best to construct a PRS for a particular phenotype. A PRS of the general form $\sum_{i=1}^{K}\hat{\beta_{i}}G_{i}$ requires specification of three things: a list of markers *G*_1_, *G*_2_, ⋯*G*_*K*_, the depth of the list or the number of markers (*K)*, and the choice of the weights $\hat{\beta_{i}}$. These choices can be based on information extracted from the latest GWAS or GWAS meta-analysis (when available), the NHGRI-EBI GWAS catalog of published results [1] (when available), or summary data for GWAS corresponding to each phenotype, e.g., from efforts that comprehensively screened the UK Biobank (UKB) phenome [8, 9]. While various methods for constructing PRS have been widely studied for predicting the primary phenotype collected through population-based sampling [6, 10], it is unknown how the different PRS will be associated with subtypes and related phenotypes and the associations PRS can help unravel across the medical phenome. The comparative performance of different PRS construction methods may depend on the phenotype of interest. For example, diseases such as depression, which are believed to involve genetic contribution of a large number of genetic variants, might perform differently than diseases such as cancer, which may involve a smaller number of causal variants. We provide important empirical results comparing different PRS construction methods in terms of their associations with secondary and related phenotypes and in terms of the associations they identify across the phenome.

In this paper, we first explore strategies for constructing a PRS using markers and weights obtained from either the latest GWAS or the NHGRI-EBI GWAS catalog that have reached genome-wide significance. We compare the PRS in terms of their performance [11] for the three most common skin cancer subtypes in the USA: basal cell carcinoma (MIM: 614740) [12], cutaneous squamous cell carcinoma [13] and melanoma (MIM: 155601) [14]. We compare the two strategies using an independent biobank of genetic, demographic, and phenotype data collected by the Michigan Genomics Initiative (MGI), a longitudinal biorepository effort within Michigan Medicine (University of Michigan) [4, 15]. Based on these results, we choose a PRS construction strategy for each skin cancer subtype for further analysis.

For the chosen PRS corresponding to each skin cancer subtype, we perform a phenome-wide association study (PheWAS) relating the PRS to the EHR-based phenome of MGI. We call such a study a PRS-PheWAS [4]. PRS-PheWAS results are then replicated using the population-based UK Biobank data. In order to identify secondary associations that are not driven by the primary phenotype, we perform an additional “exclusion” PRS-PheWAS for each skin cancer subtype in which we exclude subjects with any type of observed skin cancer [4]. These studies demonstrate similarities and differences in PheWAS results for PRS constructed for different disease subtypes, suggesting that PRS constructed for various disease subtypes can provide insight into shared and unique secondary associations. Our results further demonstrate the ability of such studies to reproduce known associations between secondary phenotypes and particular disease subtypes through use of PRS.

We then describe an approach for using PRS to more directly evaluate the shared and unique genetic architecture of disease subtypes and identify shared and unique secondary phenotype associations related to this genetic architecture. We define a new PRS for each skin cancer subtype using loci ***unique*** to that subtype’s chosen PRS. We further construct a composite PRS for general skin cancer consisting of loci ***common*** among all subtypes’ PRS. While merging distinct clinical entities into a compound PRS may seem counterintuitive in terms of specificity, such an approach may increase power to identify dermatological features through PheWAS that are shared by all three subtypes. Such features may help provide insight into the shared biological etiology and disease development across disease subtypes.

The NHGRI-EBI GWAS catalog and latest GWAS PRS construction methods are based on published GWAS studies, which only report risk variants that reached genome-wide significance (usually defined by a P-value threshold of P < 5x10^-8^). However, it is likely that there are additional risk variants below this threshold that could be associated with the trait but have not reached statistical significance [16]. Incorporating non-significant variants may conceivably improve the predictive power of a PRS but may also add additional random false positive signals, which in turn could dilute the discriminatory power of the true risk variants and diminish any predictive gain [6, 17]. To explore whether a PRS constructed using additional non-significant loci may perform differently than a PRS using only loci reaching genome-wide significance, we evaluated PRS constructed using publicly available genome-wide summary statistics from the UK Biobank at six different p-value thresholds both in terms of associations with skin cancer phenotypes and in terms of secondary phenotype associations. We further applied LDpred, a tool that adjusts GWAS summary statistics for the effects of linkage disequilibrium [7], to explore the performance of PRS incorporating the entire spectrum of available genetic information across the genome. There is extensive literature on constructing genome-wide PRS using random effects, shrinkage methods, or thresholding (our focus) [7, 18, 19], but none of these methods have been evaluated in a PheWAS setting.

In this paper, we focus our attention on skin cancer, but the approaches used in this paper can be applied to other diseases with well-defined molecular subtypes (for example, breast cancer with ER and HER2-defined subtypes). We chose to use skin cancer as a demonstrative example for a variety of reasons. First, our discovery dataset (MGI) is particularly enriched for skin cancer cases due to the strong skin cancer clinical program at Michigan Medicine and due to the high rate of surgery for skin cancer patients. MGI primarily recruits participants undergoing surgery and is therefore enriched for cancers and other medical comorbidities when compared to a general population [4]. Additionally, skin cancer has well-defined subtypes, which allows us to explore performance of subtype-specific PRS. Skin cancer also provides a setting in which there may be genetic factors uniquely related to particular subtypes as well as genetic factors that are shared risk factors for all skin cancer subtypes. The various PRS–phenotype associations identified in this paper demonstrate ways to explore shared and subtype-specific phenotypes, and this joint framework may provide an enhanced understanding of the genome x phenome landscape.

We introduce an online visual web catalog called *PRSweb* that provides PRS-PheWAS results for melanoma, basal cell carcinoma, and squamous cell carcinoma. PheWAS results are available using four different PRS construction methods explored in this paper: latest GWAS, NHGRI-EBI GWAS catalog, the UK Biobank GWAS summary statistics using different significance thresholds, and LDpred. The weights and the marker list for each PRS method can be downloaded. Furthermore, PheWAS summary statistics can be accessed from *PRSweb* (see **Web resources**), providing future investigators with readily available and useful tools to perform further analyses.

Comprehensive phenome-wide and genome-wide analyses of large biobank studies with publicly available summary statistics can be rich resources for PRS construction, especially if the trait-of-interest’s prevalence is high in the biobank. Using PRS, we can synthesize complex genetic information that can then be used to identify these shared genetic components across phenotypes. Compared to prior and existing literature, our contribution is new in four principal directions: (1) comparing various PRS construction methods in terms of their relationships with *related* EHR-derived phenotypes and subtypes (2) comparing PRS associations with secondary phenotypes across the phenome of MGI (academic medical center) and UK Biobank (population-based), (3) developing PRS-based methods for understanding the shared and unique genetic contribution across disease subtypes both in terms of disease biology and in terms of secondary phenotype associations, and (4) introducing a publicly accessible online visual catalog PRSweb to visually represent the PRS x phenome landscape and access summary data from PheWAS.

## Materials and methods

### Discovery and replication cohorts

#### MGI cohort (discovery cohort)

Participants were recruited through the Michigan Medicine health system while awaiting diagnostic or interventional procedures either during a preoperative visit prior to the procedure or on the day of the procedure that required anaesthesia. In addition to coded biosamples and secure protected health information, participants understood that all EHR, claims, and national data sources–linkable to the participant–may be incorporated into the MGI databank. Each participant donated a blood sample for genetic analysis, underwent baseline vital signs and a comprehensive history and physical assessment (also see Ethics Statement below). In the current study, we report results obtained from 30,702 unrelated, genotyped samples of recent European ancestry with available integrated EHR data (~90% of all MGI participants were inferred to be of recent European ancestry) [4].

#### UK Biobank cohort (replication cohort)

The UK Biobank is a population-based cohort collected from multiple sites across the United Kingdom and includes over 500,000 participants aged between 40 and 69 years when recruited in 2006–2010 [20]. The open access UK Biobank data used in this study included genotypes, the Ninth and Tenth Revision of the International Statistical Classification of Diseases (ICD9 and ICD10) codes, inferred sex, inferred White British ancestry, kinship estimates down to third degree, birthyear, genotype array, and precomputed principal components of the genotypes.

### Genotyping, sample quality control and imputation

#### MGI

DNA from 37,412 blood samples was genotyped on customized Illumina Infinium CoreExome-24 bead arrays and subjected to various quality control filters that resulted in a set of 392,323 polymorphic variants. Principal components and ancestry were estimated by projecting all genotyped samples into the space of the principal components of the Human Genome Diversity Project reference panel using PLINK (938 unrelated individuals) [21, 22]. Pairwise kinship was assessed with the software KING [23], and the software fastindep was used to reduce the data to a maximal subset that contained no pairs of individuals with 3rd-or closer degree relationship [24]. We also removed patients not of recent European descent from the analysis, resulting in a final sample of 30,702 unrelated subjects. Additional genotypes were obtained using the Haplotype Reference Consortium panel of the Michigan Imputation Server [25] and included over 17 million imputed variants with R^2^ ≥0.3 and minor allele frequency (MAF) ≥0.1%. Genotyping, quality control and imputation are described in detail elsewhere [4]. Table 1 provides some descriptive statistics of the MGI and UK Biobank samples.

**Table 1:** Demographics and clinical characteristics of the analytic datasets.

Characteristic	MGI	UK Biobank^a^
n	30,702	408,961
Females, n (%)	16,297 (53.1%)	221,052 (54.1%)
Mean age, years (S.D.)	54.2 (15.9)	57.7 (8.1)
Median number of visits per participant	27	not available
Median days between first and last visit	1,469	not available
Total number of ICD9 code days	3,459,331	49,085
Number of unique ICD9 codes	10,323	3,126
Median ICD9 code days per participant	58	2
Total number of ICD10 code days	1,311,264	2,764,868
Number of unique ICD10 codes	14,997	11,059
Median ICD10 code days per participant	27	6
Total number of PheWAS code days	6,367,117	3,679,624
Number of unique PheWAS codes	1,856	1,680
Median PheWAS code days per participant	94	8
n samples without skin cancer diagnosis Mean age, years (S.D.) Females, n (%)	26,199 52.6 (15.8) 14,320 (54.7%)	395,179 57.7 (8.0) 214,237 (54.2%)
n cases with skin cancer Mean age, years (S.D.) Females, n (%)	4,503 63.6 (13.3) 1,977 (43.9%)	13,782 (13,624^c^) 62.0 (6.7) 6,815 (49.4%)
n cases with melanomas of skin	1,772	2,724 (2,718^c^)
n cases with epithelial skin cancer and others^b^	3,220	11,152 (11,030^c^)
n cases with basal cell carcinoma	1,303	not available
n cases with squamous cell carcinoma	836	not available

^a^ The provided characteristics are based a subset of White British subjects of the UK Biobank Study for which phenotype data and imputed data was available. To retain as many unrelated cases as possible for each trait, a maximal set of unrelated cases was identified before choosing controls from the pool of subjects unrelated to these cases or to each other.

^b^ Original PheWAS code “172.2” description "Other non-epithelial cancer of skin".

^c^ Unrelated cases

ICD9 and ICD10: International Statistical Classification of Diseases codes (9^th^ and 10^th^ revision), MGI based on code systemts with clinical modiciations ICD9-CM and ICD10-CM; S.D. standard deviation

#### UK Biobank

The UK Biobank is a population-based cohort collected from multiple sites across the United Kingdom [20]. After quality control, we phased and imputed the 487,409 UK Biobank genotyped samples against the Trans-Omics for Precision Medicine (TOPMed) reference panel (see **Web resources**), which is composed of 60,039 multi-ethnic samples and 239,756,147 SNP and indel variants sequenced at high depth (30x). The phasing step was carried out on 81 chromosomal chunks with around 10,000 genotyped variants in each chunk using the software Eagle (with the “kbpwt” parameter set at 80,000) [26]. The imputation was carried out in 137 chromosomal chunks of around 20 Mbp in length with Mbp of total overlap on either side using the imputation tool Minimac4 (see **Web resources**). To increase computational efficiency, we imputed each of the chunks in batches of 10,000 samples at a time and then merged them back using BCFtools. Since Minimac4 imputes each sample independently, analyzing our samples in batches did not change their imputation estimates. However, this sampling would result in different imputation quality estimates for each batch, and thus we collapsed the estimates to generate imputation quality estimates across all the study samples. After imputation, we filtered out variants with estimated imputation accuracy of R^2^ < 0.1, which left us with 177,895,992 variants.

### Phenome generation

#### MGI

The MGI phenome was used as the discovery dataset and was based on ICD9 and ICD10 code data for 30,702 unrelated, genotyped individuals of recent European ancestry. These ICD9 and ICD10 codes were aggregated to form up to 1,857 PheWAS traits using the PheWAS R package (as described in detail elsewhere [4, 27]). For each trait, we identified case and control samples. To minimize differences in age and sex distributions or extreme case-control ratios as well as to reduce computational burden, we matched up to 10 controls to each case using the R package “MatchIt” [28]. Nearest neighbor matching was applied for age and PC1-4 (using Mahalanobis-metric matching; matching window caliper/width of 0.25 standard deviations) and exact matching was applied for sex and genotyping array. A total of 1,578 case-control studies with >50 cases were used for our analyses of the MGI phenome.

#### UK Biobank

The UK Biobank phenome was used as a replication dataset and was based on ICD9 and ICD10 code data of 408,961 White British [15], genotyped individuals that were aggregated to PheWAS traits in a similar fashion (as described elsewhere [9]). To remove related individuals and to retain larger sample sizes, we first selected a maximal set of unrelated cases for each phenotype (defined as no pairwise relationship of 3^rd^ degree or closer [24, 29]) before selecting a maximal set of unrelated controls unrelated to these cases. Similar to MGI, we matched up to 10 controls to each case using the R package “MatchIt” [28]. Nearest neighbor matching was applied for birthyear and PC1-4 (using Mahalanobis-metric matching; matching window caliper/width of 0.25 standard deviations) and exact matching was applied for sex and genotyping array. A total of 1,366 case-control studies with >50 cases each were used for our analyses of the UK Biobank phenome.

On average, we were able to match 9.3 controls per case in the MGI phenome and 9.9 controls per case in the UKB phenome. Additional phenotype information for MGI and UK Biobank is included in **Fig B in** S1 Text and **Tables F-H in** S1 File.

### Risk SNP selection

For each skin cancer subtype (melanoma, basal cell carcinoma, and squamous cell carcinoma), we generated four different sets of PRS: (1) based on merged summary statistics published in the NHGRI EBI GWAS catalog [1], (2) based on the latest available GWAS meta-analysis [30–32], (3) based on linkage disequilibrium (LD) clumping and p-value thresholding on publicly available GWAS summary statistics from the UK Biobank data [9], and (4) based on reweighting effect sizes of GWAS summary statistics by modeling LD with LDpred [7].

#### GWAS catalog SNP selection

We downloaded previously reported GWAS variants from the NHGRI-EBI GWAS catalog (file date: February 28, 2018) [1, 33]. None of the currently available skin cancer discovery studies included in the catalog used any subset of the MGI cohort or data from the UK Biobank. Single nucleotide polymorphism (SNP) positions were converted to GRCh37 using variant IDs from dbSNP: build 150 (UCSC Genome Browser) after updating outdated dbSNP IDs to their merged dbSNP IDs. Entries with missing risk alleles, risk allele frequencies, or odds ratios were excluded. If a reported risk allele did not match any of the reported forward strand alleles of a non-ambiguous SNP (not A/T or C/G) in the imputed genotype data (which correspond to the alleles of the imputation reference panel), we assumed minus strand designation and corrected the effect allele to its complementary base of the forward strand. Entries with a reported risk allele that did not match any of the alleles of an ambiguous SNP (A/T and C/G) in our data were excluded at this step. We only included entries with broad European ancestry (as reported by the NHGRI-EBI GWAS catalog). As a quality control check, we compared the reported risk allele frequencies (RAF) in controls with the RAF of 14,770 MGI individuals who had no cancer diagnosis (for chromosome X variants, we calculated RAF in females only). We then excluded entries whose RAF deviated more than 15%. This chosen threshold is subjective and was based on clear differentiation between correct and likely flipped alleles on the two diagonals (see **Fig A in** S1 Text) as noted frequently in GWAS meta-analyses quality control procedures [34]. No p-value threshold was applied to accommodate GWAS entries that used discovery/replication-based approaches to define statistical significance. For each analyzed cancer type, we extracted risk variants that were also present in our genotype data and estimated pairwise LD (correlation r^2^) using the allele dosages of the corresponding controls. For pairwise correlated SNPs (r^2^>0.1) or SNPs with multiple entries, we kept the SNP with the most recent publication date (and smaller *P* value, if necessary) and excluded the other (**Table I in** S1 File).

#### Selection of risk SNPs from latest GWAS

In a similar fashion, we extracted and filtered reported association signals from large GWAS meta-analyses on basal cell carcinoma [31], cutaneous squamous cell carcinoma [30] and melanoma [32] and further restricted our attention to GWAS associations with p-values < 5x10^-8^ (**Table I in** S1 File).

#### Genome-wide SNP selection of UK-Biobank-based GWAS

We obtained GWAS summary statistics for the ICD9- and ICD10-based PheWAS codes “172” (skin cancer; 13,752 cases versus 395,071 controls), “172.11” (melanoma; 2,691 cases versus 395,071 controls), and “172.2” (non-epithelial skin cancer; 11,149 cases versus 395,071 controls) from a public download [9] (see **Web resources**). These GWAS analyzed up to 408,961 White British European-ancestry samples with generalized mixed model association tests that used the saddlepoint approximation to calibrate the distribution of score test statistics and thus could control for unbalanced case-control ratios and sample relatedness [9]. For each trait, we reduced these summary statistics to SNPs that were reported with minor allele frequencies > 0.5% and were also available for the MGI data. Next, we performed LD clumping of all variants with p-values < 5x10^-4^ using the imputed allele dosages to obtain independent risk SNPs (LD threshold of r^2^ > 0.1 and a maximal SNP distance of 1 Mb). We limited the LD calculations to 10,000 randomly selected, unrelated, White British individuals to reduce the computational burden. Finally, we created subsets of these independent SNPs with p-values <5x10^-9^, <5x10^-8^, <5 x10^-7^, <5x10^-6^, <5x10^-5^, and <5x10^-4^ (**Table J in** S1 File).

As an alternative to the above LD clumping and p-value-thresholding approach of genome-wide PRS, we used the software package LDpred [7] to reweight the effect size of each variant of GWAS summary statistics by using a prior on effect sizes and LD information from a reference panel. We randomly selected 5,000 unrelated, White British samples of the imputed UK Biobank genotype data as the LD reference panel. We used the summary statistics of the UK Biobank-based GWAS on skin cancer (PheWAS code “172”) and melanoma (PheWAS code “172.11) and applied LDpred’s default filters (SNPs only; overlap between summary statistics, LD reference panel and target panel; MAF > 1%; non-ambiguous allele combinations), which resulted in 6.4 million variants for each GWAS. For each of the GWAS summary statistics, we ran LDpred with an LD radius of 2,800 SNPs, which corresponds to an average 1 Mb window, and modelled six proportions of causal SNPs in the prior (100%, 10%, 1%, 0.1%, 0.01%, and 0.001%) to obtain six genome-wide SNP sets with LDpred-reweighted effect sizes.

### Construction of the polygenic risk scores

For each of the obtained SNP sets for each trait, we constructed a PRS as the sum of the allele dosages of risk increasing alleles of the SNPs weighted by their reported or reweighted log odds ratios. Restated, the PRS for subject j in MGI was of the form PRS_*j*_ = Σ_*i*_*β*_*i*_*G*_*ij*_ where *i* indexes the included loci for that trait, *β*_*i*_ is the log odds ratios retrieved from the external GWAS summary statistics for locus *i*, and *G*_*ij*_ is a continuous version of the measured dosage data for the risk allele on locus *i* in subject *j*. The PRS variable was created for each MGI and UKB participant. For comparability of effect sizes corresponding to the continuous PRS across cancer traits and PRS construction methods, we transformed each PRS of the corresponding analytical data set to the standard Normal distribution using “ztransform” in the R package “GenABEL” [35].

### Statistical analysis

In this study, we first constructed PRS for skin cancer subtypes using either the latest GWAS or the corresponding entries of the GWAS catalog. To compare the association between PRS and skin cancer phenotypes across different PRS construction methods, we fit the following model for each PRS and skin cancer phenotype:

$$\begin{array}{l} \text{logit}(\text{P}(\text{Phenotype}\;\text{is}\;\text{present}|\text{PRS},\text{Age},\text{Sex},\text{Array},\text{PC}))\\ =\beta_{0}+\beta_{PRS}\text{PRS}+\beta_{Age}\text{Age}+\beta_{Sex}\text{Sex}+\beta_{Array}\text{Array}+\beta \;\text{PC}, \end{array}$$

where the PCs were the first four principal components obtained from the principal component analysis of the genotyped GWAS markers and where “Array” represents the genotyping array. Our primary interest is *β*_*PRS*_, while the other factors (Age, Sex and PC) were included to address potential residual confounding and do not provide interpretable estimates due to the preceding application of case-control matching. Firth’s bias reduction method was used to resolve the problem of separation in logistic regression (Logistf in R package “EHR”) [36–38], a common problem for binary or categorical outcome models when a certain part of the covariate space has only one observed value of the outcome, which often leads to very large parameter estimates and standard errors.

We then evaluated each PRS’s (1) ability to discriminate between cases and controls by determining the area under the receiver-operator characteristics (ROC) curve (AUC) using R package “pROC” [39]; (2) calibration using Hosmer-Lemeshow Goodness of Fit test in the R package “ResourceSelection” [40, 41]; and (3) accuracy with the Brier Score in the R package “DescTools” [42]. These evaluations did not adjust for additional covariates. These metrics were estimated using roughly 2/3 of the matched data as a test set after fitting the above model on the remaining 1/3 of matched data, which we will refer to as the training data. We used these metrics and the logistic regression results to choose a PRS construction method to use for each skin cancer subtype moving forward. We compare these measures for various PRS-phenotype relationships for each phenotype separately, so the comparative performance of these measures is not biased by the different case-control sampling. To explore the impact of incorporating non-significant loci into the PRS construction, we further performed the above analyses with PRS constructed using UK Biobank GWAS summary statistics with different p-value thresholds. Similarly, we compared the LDpred-based PRS that assumed six different fractions of causal variants (non-zero effects) in the prior: 100%, 10%, 1%, 0.1%, 0.01%, and 0.001%. For LDpred comparisons we also report Nagelkerke’s pseudo-R^2^ to be consistent with the LDpred workflow [7].

Using the chosen PRS for each subtype, we conducted two PheWAS to identify other phenotypes associated with the PRS first for the 1,578 phenotypes in MGI and then for the 1,366 phenotypes from UK Biobank. To evaluate PRS-phenotype associations, we conducted Firth bias-corrected logistic regression by fitting a model of the above form for each phenotype and data source. Age represents the birth year in UK Biobank. To adjust for multiple testing, we applied the conservative phenome-wide Bonferroni correction according to the analyzed PheWAS codes (n_MGI_ = 1,578 or n_UK Biobank_ = 1,366). In Manhattan plots, we present–log10 (*p*-value) corresponding to tests of *H*_0_: *β*_*PRS*_ = 0. Directional triangles on the PheWAS plot indicate whether a phenome-wide significant trait was positively (pointing up) or negatively (pointing down) associated with the PRS.

To investigate the possibility of the secondary trait associations with PRS being completely driven by the primary trait association, we performed a second set of PheWAS after excluding individuals affected with the primary or related cancer traits for which the PRS was constructed, referred to as “exclusion PRS PheWAS” as described previously [4]. We then constructed new PRS scores representing shared and subsite-unique genetic components and performed a PheWAS for each.

To evaluate the impact of the matching in the PRS PheWAS and exclusion PRS PheWAS analyses in more concrete terms, we performed sensitivity analyses in which we conducted the PheWAS analyses using the unmatched data.

To evaluate how well prior presence of a secondary diagnosis can identify subjects with increased risk of developing skin cancer, we created a binary variable taking the value 1 if a given subject (1) was diagnosed with the secondary diagnosis and then diagnosed with skin cancer at least 365 days after or (2) was diagnosed with the secondary diagnosis and never diagnosed with skin cancer. We then fit a Firth bias-corrected logistic regression of the following form:

$$\begin{array}{l} \text{logit}(\text{P}(\text{Primary}\;\text{phenotype}\;\text{is}\;\text{present}|\text{Predictor},\text{Age},\text{Sex},\text{Array},\text{PC}))\\ =\beta_{0}+\beta_{PRS}I(\text{Secondary}\;\text{non}\;\text{skin}\;\text{cancer}\;\text{trait})+\beta_{Age}\text{Age}+\beta_{Sex}\text{Sex}+\beta_{Array}\text{Array}+\beta \;\text{PC} \end{array}$$

where Array and PC were defined as before. Unless otherwise stated, analyses were performed using R 3.4.4 [43].

### Development of an online visual catalog: *PRSweb*

The online open access visual catalog *PRSweb* available at http://statgen.github.io/PRSweb was implemented using “Pandas”, a Data Analysis Library, which offers high level performance for large data structures and data analysis in the Python3 environment [44]. In combination with “Jinja2”, a templating language for Python, and “Bootstrap”, a Cascading Style Sheets (CSS) framework (see Web resources), static HTML files were compiled and allow easy and fast hosting of all PRS-PheWAS results. The interactive plots are drawn with the JavaScript library “LocusZoom.js” (see Web resources) which offers dynamic plotting, automatic plot sizing and label positioning.

### Ethics statement

Data were collected according to Declaration of Helsinki principles. MGI study participants’ consent forms and protocols were reviewed and approved by the University of Michigan Medical School Institutional Review Board (IRB ID HUM00099605 and HUM00155849). Opt-in written informed consent was obtained.

## Results

### Assessing various PRS construction methods

We first explored the comparative performance of various PRS construction strategies in terms of the resulting PRS associations with related phenotypes in the skin cancer setting. Table 2 provides the results.

**Table 2:** Associations of constructed PRS with skin cancer traits in MGI.

PRS (Number of SNPs)		Skin cancer n = 4,503 (1,501 / 3,002)^d^	Melanoma n = 1,896 (617 / 1,279)^d^	Basal cell carcinoma n = 1,303 (419 / 884)^d^	Squamous cell carcinoma n = 836 (273 / 563)^d^
*PRS based on GWAS catalog*					
Melanoma (29)	PRS OR^a^ P-value^a^ AUC^b^ HL χ^2^, P-value ^c^ Brier Score	1.3 (1.26,1.34) 2.7x10^-53^ 0.57 (0.56,0.58) 6.8, 0.56 0.14	**1.48 (1.41,1.56)** **1.3x10**^**-56**^ **0.61 (0.59,0.62)** **7.6, 0.47** **0.093**	1.3 (1.23,1.38) 7.3x10^-19^ 0.58 (0.56,0.6) 12, 0.13 0.093	1.23 (1.14,1.32) 4.3x10^-8^ 0.55 (0.53,0.58) 2.2, 0.97 0.091
Basal cell carcinoma (32)	PRS OR^a^ P-value^a^ AUC^b^ HL χ^2^, P-value ^c^ Brier Score	1.32 (1.27,1.36) 8x10^-60^ 0.58 (0.56,0.59) 9.7, 0.29 0.14	1.31 (1.25,1.37) 7.2x10^-28^ 0.58 (0.56,0.59) 13, 0.1 0.093	**1.65 (1.56,1.75)** **3.6x10**^**-65**^ **0.64 (0.62,0.66)** **15, 0.066** **0.091**	1.32 (1.23,1.42) 1.4x10^-14^ 0.58 (0.55,0.6) 12, 0.17 0.091
Squamous cell carcinoma (18)	PRS OR^a^ P-value^a^ AUC^b^ HL χ^2^, P-value ^c^ Brier Score	1.25 (1.21,1.3) 4.8x10^-42^ 0.56 (0.55,0.57) 8.1, 0.42 0.14	1.32 (1.26,1.39) 5.1x10^-31^ 0.58 (0.56,0.59) 6.2, 0.63 0.093	1.35 (1.28,1.43) 7.9x10^-26^ 0.58 (0.56,0.6) 5.9, 0.66 0.093	1.26 (1.17,1.35) 1.8x10^-10^ 0.55 (0.53,0.58) 18, 0.025 0.091
*PRS based on latest GWAS*					
Melanoma (20)	PRS OR^a^ P-value^a^ AUC^b^ HL χ^2^, P-value ^c^ Brier Score	1.31 (1.27,1.36) 3.5x10^-55^ 0.57 (0.56,0.58) 4.9, 0.76 0.14	1.49 (1.42,1.57) 1.2x10^-55^ 0.6 (0.59,0.62) 13, 0.13 0.093	1.39 (1.3,1.47) 7.9x10^-27^ 0.59 (0.58,0.61) 16, 0.04 0.092	1.25 (1.16,1.34) 4x10^-9^ 0.55 (0.53,0.58) 7.6, 0.47 0.091
Basal cell carcinoma (28)	PRS OR^a^ P-value^a^ AUC^b^ HL χ^2^, P-value ^c^ Brier Score	1.32 (1.28,1.37) 5.8x10^-61^ 0.58 (0.57,0.59) 5.9, 0.66 0.14	1.33 (1.27,1.4) 5.7x10^-32^ 0.58 (0.57,0.6) 20, 0.012 0.093	1.62 (1.53,1.71) 2.8x10^-60^ 0.63 (0.61,0.65) 6.5, 0.59 0.091	1.34 (1.25,1.44) 1.2x10^-15^ 0.58 (0.55,0.6) 15, 0.052 0.091
Squamous cell carcinoma (10)	PRS OR^a^ P-value^a^ AUC^b^ HL χ^2^, P-value ^c^ Brier Score	1.34 (1.3,1.38) 1.1x10^-70^ 0.58 (0.57,0.6) 13, 0.12 0.14	1.42 (1.36,1.49) 6.1x10^-51^ 0.59 (0.58,0.61) 14, 0.082 0.093	1.47 (1.4,1.56) 1.8x10^-43^ 0.61 (0.59,0.63) 7.1, 0.53 0.092	**1.4 (1.31,1.5)** **2.1x10**^**-21**^ **0.59 (0.56,0.61)** **6.4, 0.61** **0.091**

^a^ Association of each cancer with continuous PRS that were transformed to standard normal distribution. Point estimates, 95% confidence intervals and P- values are obtained by fitting Firth’s bias-corrected logistic regression adjusted for age, sex, batch and PC1-4 to the full data.

^b^ Area under the curve of the receiver operating characteristic (ROC) curve with 95% confidence intervals calculated using the test data after fitting a model with the training data.

^c^ Hosmer-Lemeshow Goodness-of-Fit test for the test data after fitting a model with the training data

^d^ Number of cases in training / test set

#### PRS performance across related phenotypes

Using both the GWAS catalog and latest GWAS construction methods, the melanoma PRS was most strongly associated with the melanoma phenotype compared to the other phenotypes (based on the odds ratio). In particular, both melanoma PRS are more strongly related to the melanoma phenotype than the overall skin cancer phenotype, indicating that the reduced sample size associated with a more granular phenotype definition did not negatively impact the PRS specificity. Similarly, the two basal cell carcinoma PRS were most strongly associated with the basal cell carcinoma skin cancer subtype. Unlike the other cancer subtypes, the squamous cell carcinoma PRS did not appear to be most strongly associated with the squamous cell carcinoma phenotype. Instead, it was most strongly associated with basal cell carcinoma phenotype.

#### Comparisons across PRS construction methods

For each skin cancer phenotype, we compared the PRS-phenotype associations for various PRS. **Overall Skin Cancer**: PRS defined using different skin cancer subtypes had similar performance in terms of association with and discrimination for the overall skin cancer phenotype. By “discrimination,” we refer to the ability of the PRS to distinguish cases and controls, which is measured by AUC. **Melanoma**: For the melanoma PRS, the GWAS catalog method and the latest GWAS method produced similar performance in terms of AUC, OR, Hosmer-Lemeshow goodness of fit, and Brier score. For example, the AUC for melanoma for the GWAS catalog melanoma PRS was 0.61 (95% CI, [0.59, 0.62]). The corresponding AUC for the latest GWAS method was 0.60 (95% CI, [0.59, 0.62]). The two melanoma PRS out-performed PRS for the other skin cancer subtypes in terms of association with and discrimination for the melanoma phenotype. **Fig C in** S1 Text compares PRS weights to corresponding SNP-melanoma associations in MGI and UK Biobank. **Basal Cell Carcinoma**: As with melanoma, the basal cell carcinoma PRS produced similar results under the GWAS catalog and latest GWAS construction methods. The basal cell carcinoma AUC under both the GWAS catalog and latest GWAS methods were 0.64 (95% CI, [0.62, 0.66]) and 0.63 (95% CI, [0.61, 0.65]), respectively. The OR values and Brier score values were nearly identical, and neither approach produced evidence of lack of fit based on the Hosmer-Lemeshow statistic. **Squamous Cell Carcinoma**: The squamous cell carcinoma latest GWAS-based PRS was more strongly associated with the basal cell carcinoma phenotype than the squamous cell carcinoma GWAS catalog PRS, with an odds ratio of 1.4 (95% CI, [1.31, 1.5]). The squamous cell carcinoma phenotype using the GWAS catalog method produced a lower AUC (0.55, 95% CI [0.53, 0.58]) compared to the latest GWAS method (0.59, 95% CI [0.56, 0.61]). While a difference of 0.04 may not seem like a large difference in AUC in other applications, any improvement in AUC for PRS associations with observed phenotypes may be considered appreciable [45]. These two methods produced similar Brier scores.

Using the above comparisons between the various PRS, we chose a single PRS construction method for each skin cancer subtype to use in subsequent analyses. For melanoma and basal cell carcinoma, we chose the GWAS catalog method. While the GWAS catalog and latest GWAS methods were very similar for these two subtypes, we chose to pursue the GWAS catalog PRS for future analysis due to the larger number of loci for these PRS (29 vs 20 for melanoma and 32 vs 28 for basal cell carcinoma). We choose the latest GWAS method for squamous cell carcinoma due to its improved AUC and stronger OR compared to the GWAS catalog method. We will denote the chosen PRS for melanoma, basal cell carcinoma, and squamous cell carcinoma as mPRS, bPRS, and sPRS respectively.

### PheWAS using the chosen PRS in MGI

**Fig 1:**
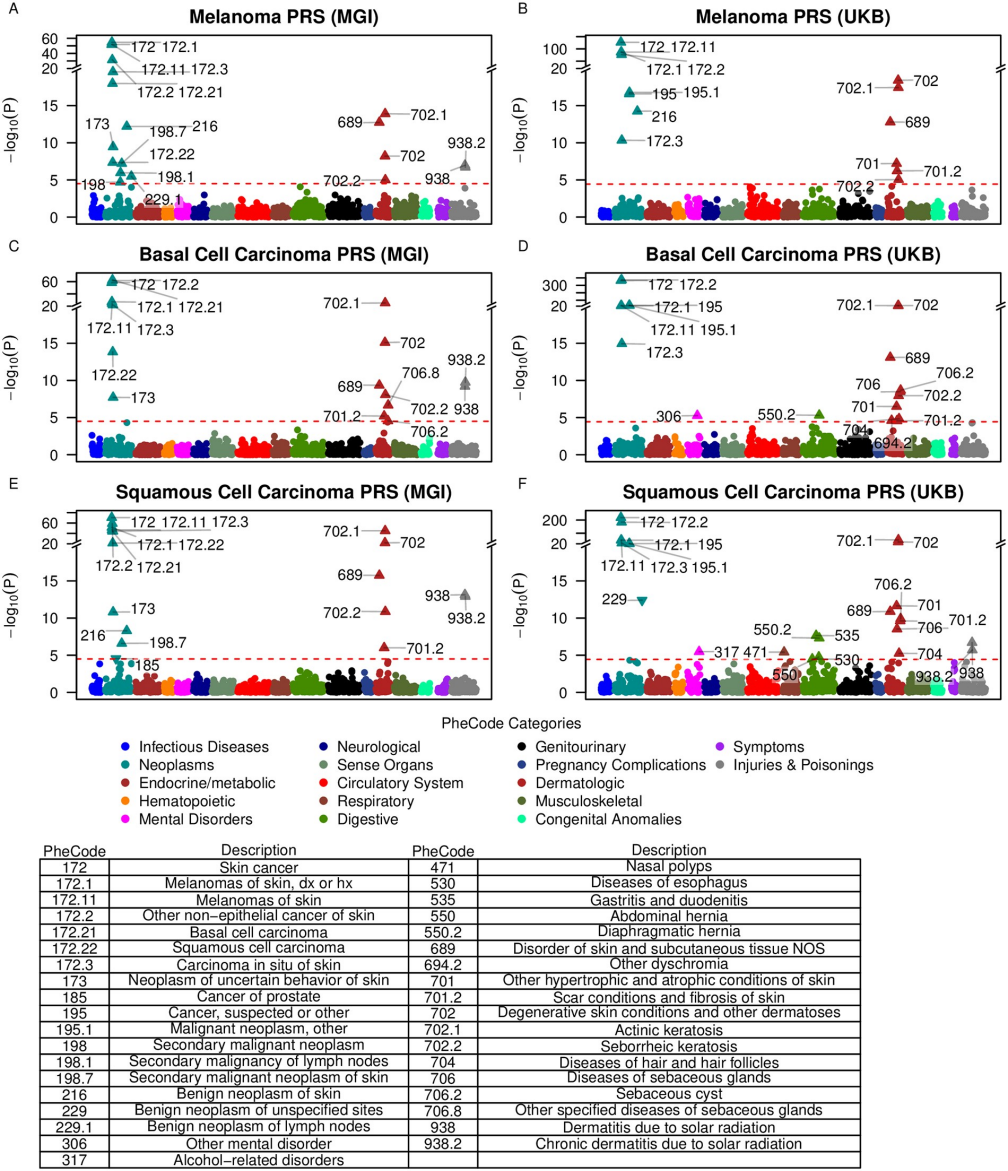
PRS-PheWAS in MGI and UKB phenomes. The horizontal line indicates phenome-wide significance. Phenome-wide significant traits are indicated by PheCodes with their description listed below. Directional triangles indicate whether a phenome-wide significant trait was positively (pointing up) or negatively (pointing down) associated with the PRS.

Using each of the chosen PRS described above (mPRS, bPRS, and sPRS), we tested the association between each PRS and each of the 1,578 constructed phenotypes in MGI. For each PRS, the strongest associations were observed with dermatologic neoplasms that included overall skin cancer, melanoma, “other non-epithelial cancer of skin” (the PheWAS parent category of basal and squamous cell carcinoma), and carcinoma in situ of skin. In addition, secondary dermatologic traits such as actinic keratosis (with parent category “degenerative skin conditions and other dermatoses”), chronic dermatitis due to solar radiation (with parent category “dermatitis due to solar radiation”), and seborrheic keratosis were found to be associated with all three PRS (Fig 1 and **Table K in** S1 File). “Diseases of sebaceous glands”, “sebaceous cyst”, and “scar conditions and fibrosis of skin” were associated with bPRS. mPRS was most strongly associated with the melanoma phenotype (OR 1.48, 95% CI [1.41, 1.56]), while bPRS was most strongly associated with basal cell carcinoma (OR 1.65, 95% CI [1.56, 1.75]) followed closely by “other non-epithelial cancer of the skin” (OR 1.39, 95% CI [1.34, 1.44]). sPRS was most strongly associated with overall skin cancer (OR 1.34, 95% CI [1.3, 1.38]). The OR of all these phenotypes indicated an increased risk for primary and secondary traits with increasing PRS.

### Validation of PRS-PheWAS in UK Biobank

To substantiate the detected dermatologic associations, we reiterated the association screen of the three PRS in the matched phenome of the population-based UK Biobank data set (Fig 1). In general, stronger evidence for association was found in UKB compared to MGI. This may be driven by the larger sample sizes, e.g. a total of 13,623 skin cancer cases versus 4,503 in MGI. In the UK Biobank phenome, the large majority of the previous associations with dermatologic neoplasms were validated with the exception of the trait “dermatitis due to solar radiation”, which had substantially fewer cases in UKB compared to MGI (390 versus 2,959 cases). Unlike MGI, all three PRS were significantly associated (at the phenome-wide level) with “cancer, suspected or other” and “malignant neoplasm, other.” bPRS and sPRS were both associated with “diseases of the sebaceous glands” and “sebaceous cyst.”

### Exclusion PheWAS using the chosen PRS in MGI

In order to explore whether the identified PRS-phenotype associations were driven by the primary trait used to define the PRS (for example, as a side effect of treatment given after diagnosis with the primary trait), we performed a PheWAS for each PRS in which we excluded subjects who were cases for the primary trait or other skin cancer subtypes [4]. Results are shown in **Table K in** S1 File and **Fig D in** S1 Text. Actinic keratosis, a skin condition believed to be a precursor to non-melanoma skin cancers, remained significantly associated with the squamous cell carcinoma PRS in MGI and all three PRS in UK Biobank [46–48]. No other phenotypes were significant for MGI. “Sebaceous cyst” and its parent category “diseases of the sebaceous gland” were significant in the main UK Biobank PheWAS and remained significantly associated with basal cell carcinoma PRS and squamous cell carcinoma PRS in UK Biobank in the Exclusion PheWAS. **Appendix 1 in** S1 Text provides additional information on a sub-analysis of actinic keratosis as a predictor for future skin cancer.

### PRS-PheWAS for shared and unique loci across skin cancer subtypes

**Fig 2:**
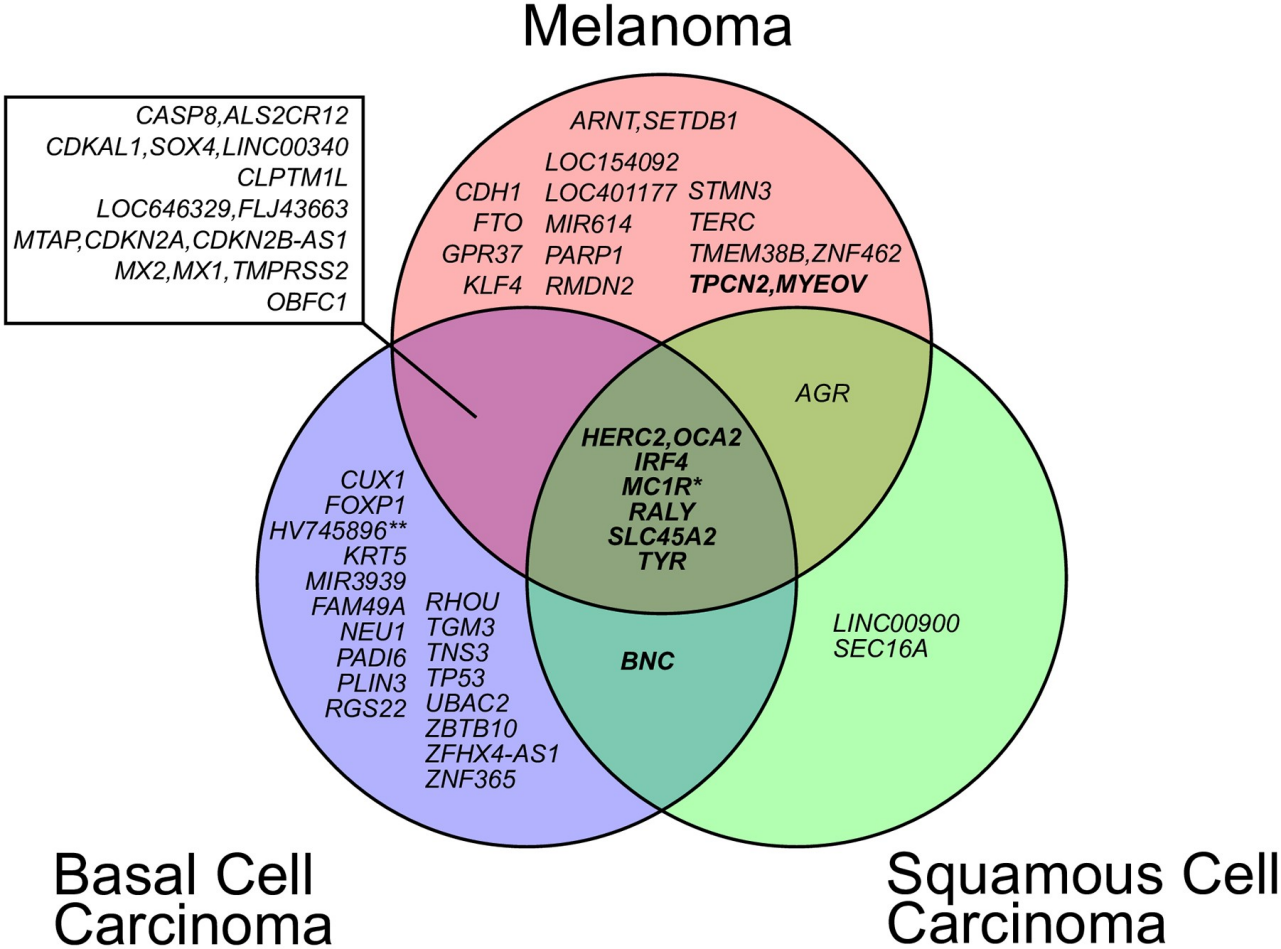
Overlap between the three skin cancer trait loci. Reported risk SNPs within 1 Mb were merged into the same locus. Loci that were also reported to be associated with skin tanning ability are highlighted in bold. Loci were named according to the closest RefSeq genes (except *M1CR* a 385 kb locus with 16 RefSeq genes and *HV745896* named after a nearby, uncurated mRNA sequence).

In the PRS-PheWAS analyses, we note a striking overlap in the secondary dermatological traits significantly associated with each of the three PRS (mPRS, bPRS, sPRS). One potential explanation for this is that subjects may have more screening after an initial skin cancer diagnosis. Indeed, many subjects have multiple skin cancer diagnoses (**Fig F in** S1 Text). Fig 2 shows the number of risk loci shared by different PRS. Six risk loci are shared between the mPRS, bPRS, and sPRS.

This observation inspired a follow-up exploration in which we defined a PRS for each cancer subtype using the loci unique to that subtype’s chosen PRS. We call these new PRS scores mPRS-u, bPRS-u, and sPRS-u, which reflect the unique loci in the PRS for melanoma, basal cell carcinoma, and squamous cell carcinoma respectively. We also define a PRS consisting of all loci shared across the three skin cancer subtypes, which we call the shared PRS.

**Table C in** S1 Text shows the association between the various constructed PRS and the skin cancer phenotypes. As with mPRS, mPRS-u was most strongly associated with the melanoma phenotype. The bPRS-u score was similarly most strongly associated with basal cell carcinoma. We note that the melanoma AUC for the mPRS score was 0.61 (95% CI, [0.59, 0.62]) and is only 0.54 (95% CI, [0.52, 0.56]) for the mPRS-u score. Similarly, the basal cell carcinoma AUC for the bPRS score was 0.64 (95% CI, [0.62, 0.66]) and is only 0.57 (95% CI, [0.55, 0.59]) for the bPRS-u score. The sPRS-u score is not more strongly associated with the squamous cell carcinoma phenotype than the other skin cancer subtypes. For this reason, we do not include this PRS in further analyses. The shared PRS constructed as the unweighted sum of risk alleles of loci present in all three PRS scores (mPRS, bPRS, and sPRS) is more strongly associated with all three subtype phenotypes than the overall skin cancer phenotype.

**Fig H in** S1 Text shows PRS-PheWAS results using mPRS-u and bPRS-u. The scores again reveal their subtype specificity, while no notable secondary associations were observed. Although not shown here, additional exploration into the loci identified uniquely for each subtype, e.g. via pathway enrichment analyses, may provide some insight into subtype-specific biological mechanisms. **Fig I in** S1 Text shows PRS-PheWAS results for the shared PRS. Most strikingly, the shared skin cancer PRS was associated with the top skin cancer and dermatologic traits that were previously found to be associated with the three partially overlapping PRS constructs, suggesting that a shared genetic risk may be driving many of these secondary associations. These six underlying loci (*HERC2* [MIM 605837] /*OCA2* [MIM 611409], *IRF4* [MIM 601900], *MC1R* [MIM 155555], *RALY* [MIM 614663], *SLC45A2* [MIM 606202] and *TYR* [MIM 606933]) were previously found to be associated not only with skin cancer traits, but also with pigmentation traits of skin, eyes and hair (Fig 2; MIM 266300) [31, 49–68].

One of these pigmentation traits, skin tanning ability, the tendency of skin to sunburn rather than to suntan, is a well-known risk factor for all skin cancer traits [68, 69]. A PRS based on the independent risk variants of a recent GWAS meta-analysis on skin tanning ability [69] was strongly associated with overall skin cancer, melanoma, basal cell carcinoma, and squamous cell carcinoma and even outperformed the constructed PRS in some cases (**Table C in** S1 Text). Furthermore, the skin tanning ability PRS PheWAS identified a very similar set of traits as the shared skin cancer PRS, but in general displayed stronger associations (**Fig I in** S1 Text).

### PRS construction based on UK Biobank summary statistics at different depths

To explore whether a PRS incorporating non-significant loci will outperform a PRS incorporating only significant loci, we constructed PRS using loci related to the phenotype at six different p-value thresholds based on publicly available GWAS summary statistics from the UK Biobank. Larger p-values indicate greater SNP depth (with more SNPs being incorporated into the PRS).

The ICD-code-based collection of UK Biobank GWAS results did not include basal cell carcinoma or squamous cell carcinoma subtypes; rather, it included only the merged trait ‘non-epithelial cancer of skin’ (**Fig B in** S1 Text). Thus, we limited our assessment of the summary statistics to the overall skin cancer GWAS and the melanoma GWAS (**Table J in** S1 File).

**Table D in** S1 Text provides the results. As with the other PRS construction methods, the melanoma PRS was most strongly associated with the melanoma phenotype for all p-value cutoffs except 5x10^-4^. For this p-value cutoff, the melanoma PRS had similar AUC and OR for the melanoma and basal cell carcinoma phenotypes. This p-value cutoff represents the least conservative inclusion cutoff with 1,193 included loci, and its results indicated that inclusion of too many suggestive SNPs at lower thresholds may reduce PRS performance. However, we also note that the most conservative cutoff (5x10^-9^) produced a PRS that was based on only six loci, which had a weaker OR and AUC compared to other PRS created with less stringent cutoffs. The best performance in terms of AUC and OR relating to the melanoma phenotype were observed for p-value thresholds 5x10^-7^ and 5x10^-8^, which included 13 and 9 loci respectively. The small number of loci identified by this method at more conservative p-value cutoffs may be driven by the lower sample size for melanoma in the UK Biobank compared to the published melanoma GWAS meta-analyses (n cases = 2,691 and n cases = 6,628 respectively). We note that the melanoma PRS constructed using the UK Biobank summary statistics produced lower AUC across all p-value thresholds than was seen for the latest GWAS and GWAS catalog PRS construction methods.

Among the skin cancer subtypes, the PRS constructed for overall skin cancer was most strongly associated with basal cell carcinoma across all p-value thresholds, with odds ratios ranging from 1.4 (95% CI [1.32, 1.48]) to 1.64 (95% CI [1.55, 1.74]). Among the PRS, the overall skin cancer PRS had the greatest discrimination for the overall skin cancer phenotype. Overall skin cancer and melanoma PRS had similar performance in terms of discrimination for the melanoma phenotype across various depths. The overall skin cancer PRS tended to be more strongly associated with and have similar or slightly better discrimination for the overall skin cancer phenotype compared to the melanoma PRS, indicating that the overall skin cancer PRS was more accurate at predicting the overall skin cancer phenotype than the melanoma PRS. The overall skin cancer PRS had very similar association with and discrimination abilities for the overall skin cancer phenotype across all p-value thresholds except the least conservative (p = 5x10^-4^), for which the AUC and odds ratio were smaller. Overall, the highest AUCs and strongest OR signals for both PRS and all skin cancer phenotypes were found at depths of 5x10^-7^ and 5x10^-8^.

In addition to associations with the primary and related phenotypes, we explored associations between PRS constructed at various UK Biobank summary statistic depths and secondary phenotypes. **Fig J** (overall skin cancer) and **Fig K** (melanoma) **in** S1 Text show PRS-PheWAS results in MGI using PRS constructed at different depths. Depths of 5x10^-7^ and 5x10^-8^ produced very similar results, and other depths tended to identify fewer phenotypes associated with the corresponding PRS. Phenotypes that were associated with the PRS at other depths had weaker associations. PRS-PheWAS using the melanoma PRS and the overall skin cancer PRS produced somewhat different results. For example, the melanoma PRS at different depths did not identify strong associations with “diseases of sebaceous glands”, which is similar to previous PRS-PheWAS results for mPRS in MGI and UKB. In contrast, the overall skin cancer PRS did identify associations with “diseases of sebaceous glands” or its subcategories for all depths except 5x10^-5^ and 5x10^-4^. **Fig L in** S1 Text provides some additional information about the impact of depth on p-values for selected secondary associations.

### PRS construction using LDpred

We evaluated the performance of PRS constructed using the LDpred algorithm, which incorporates millions of SNPs into the PRS definition. **Table E in** S1 Text provides results. For the overall skin cancer PRS, a modelled 1% proportion of causal variants produced the best results in terms of AUC and OR with respect to the overall skin cancer phenotype (OR 1.30, 95% CI [1.26, 1.35]) and AUC 0.58, 95% CI [0.56, 0.60]). For the melanoma PRS, a modelled 0.001% proportion of causal variants produced the best results with respect to melanoma (OR 1.42, 95% CI [1.36, 1.49]) and AUC 0.60, 95% CI [0.58, 0.62]). This LDpred melanoma PRS performed slightly better compared to the melanoma PRS constructed using UK Biobank summary statistics at a 5x10^-8^ depth in terms of associations with the primary phenotype. Using the PRS with a percentage of assumed causal variants producing the best pseudo R^2^ statistic from **Table E in** S1 Text, we performed a PRS-PheWAS as shown in **Figure M in** S1 Text.

Table 3 summarizes the secondary phenotypes significantly associated with various PRS at the phenome-wide level. Many general skin cancer phenotypes are strongly associated with nearly all PRS. In particular, actinic keratosis and dermatitis due to solar radiation are associated with PRS for all three disease. In contrast, sebaceous cysts and “diseases of sebaceous glands” are strongly associated with PRS for basal cell carcinoma and squamous cell carcinoma but not with PRS for melanoma.

**Table 3:** Phenome-wide significant phenotypes in MGI identified using various PRS construction strategies^a^.

		Melanoma									Basal cell carcinoma		Squamous cell carcinoma	
Method		PRS Constructed Using UK Biobank Summary Statistics^b^						LDpred	mPRS	mPRS	bPRS	bPRS	sPRS	sPRS
Data Source		MGI						MGI	MGI	UKB	MGI	UKB	MGI	UKB
Phenotype	PheCode	5x10^-9^	5x10^-8^	5x10^-7^	5x10^-6^	5x10^-5^	5x10^-4^	0.001%^c^						
Melanomas of skin	172.11	*	*	*	*	*	*	*	*	*	*	*	*	*
Melanomas of skin, dx or hx	172.1	*	*	*	*	*		*	*	*	*	*	*	*
Skin cancer	172	*	*	*	*	*		*	*	*	*	*	*	*
Other non-epithelial cancer of skin	172.2	*	*	*	*	*		*	*	*	*	*	*	*
Carcinoma in situ of skin	172.3	*	*	*	*	*		*	*	*	*	*	*	*
Basal cell carcinoma	172.21	*	*	*	*	*		*	*	n/a	*	n/a	*	n/a
Actinic keratosis	702.1	*	*	*	*			*	*	*	*	*	*	*
Benign neoplasm of skin	216	*	*	*	*			*	*	*			*	
Squamous cell carcinoma	172.22	*	*	*	*			*	*	n/a	*	n/a	*	n/a
Secondary malignant neoplasm of skin	198.7	*	*	*	*			*	*				*	
Disorder of skin and subcutaneous tissue NOS	689		*	*	*			*	*	*	*	*	*	*
Benign neoplasm of lymph nodes	229.1		*	*	*			*	*					
Neoplasm of uncertain behavior of skin	173		*	*	*			*	*		*		*	
Degenerative skin conditions & other dermatoses	702		*	*				*	*	*	*	*	*	*
Secondary malignancy of lymph nodes	198.1		*	*				*	*					
Dermatitis due to solar radiation	938		*	*				*	*		*		*	*
Chronic dermatitis due to solar radiation	938.2		*	*				*	*		*		*	*
Benign neoplasm of unspecified sites	229		*	*				*						*
Secondary malignant neoplasm	198		*	*				*	*					
Scar conditions and fibrosis of skin	701.2									*	*	*	*	*
Seborrheic keratosis	702.2								*	*	*	*	*	
Sebaceous cyst	706.2										*	*		*
Malignant neoplasm, other	195.1									*		*		*
Cancer, suspected or other	195									*		*		*
Other hypertrophic and atrophic conditions of skin	701									*		*		*
Diseases of sebaceous glands	706											*		*
Diseases of hair and hair follicles	704											*		*
Diaphragmatic hernia	550.2											*		*
# SNPs:		6	9	13	27	156	1193	6.4x10^6^	29	29	32	32	18	18

* Indicates phenotype that reached phenome-wide significance in the corresponding PRS-PheWAS

^a^ Including only diseases identified in at least two PRS-PheWAS

^b^ Evaluated at different depths (p-value thresholds indicated below)

^c^ Modelled proportion of causal variants

Notes: mPRS, bPRS, and sPRS: chosen PRS for melanoma, basal cell carcinoma, and squamous cell carcinoma. mPRS and bPRS are based on GWAS catalog entries while sPRS is based on the single, latest GWAS results [30]; n/a: not available in ICD-based phenome of the UK Biobank.

### Online visual catalog: *PRSweb*

**Fig 3:**
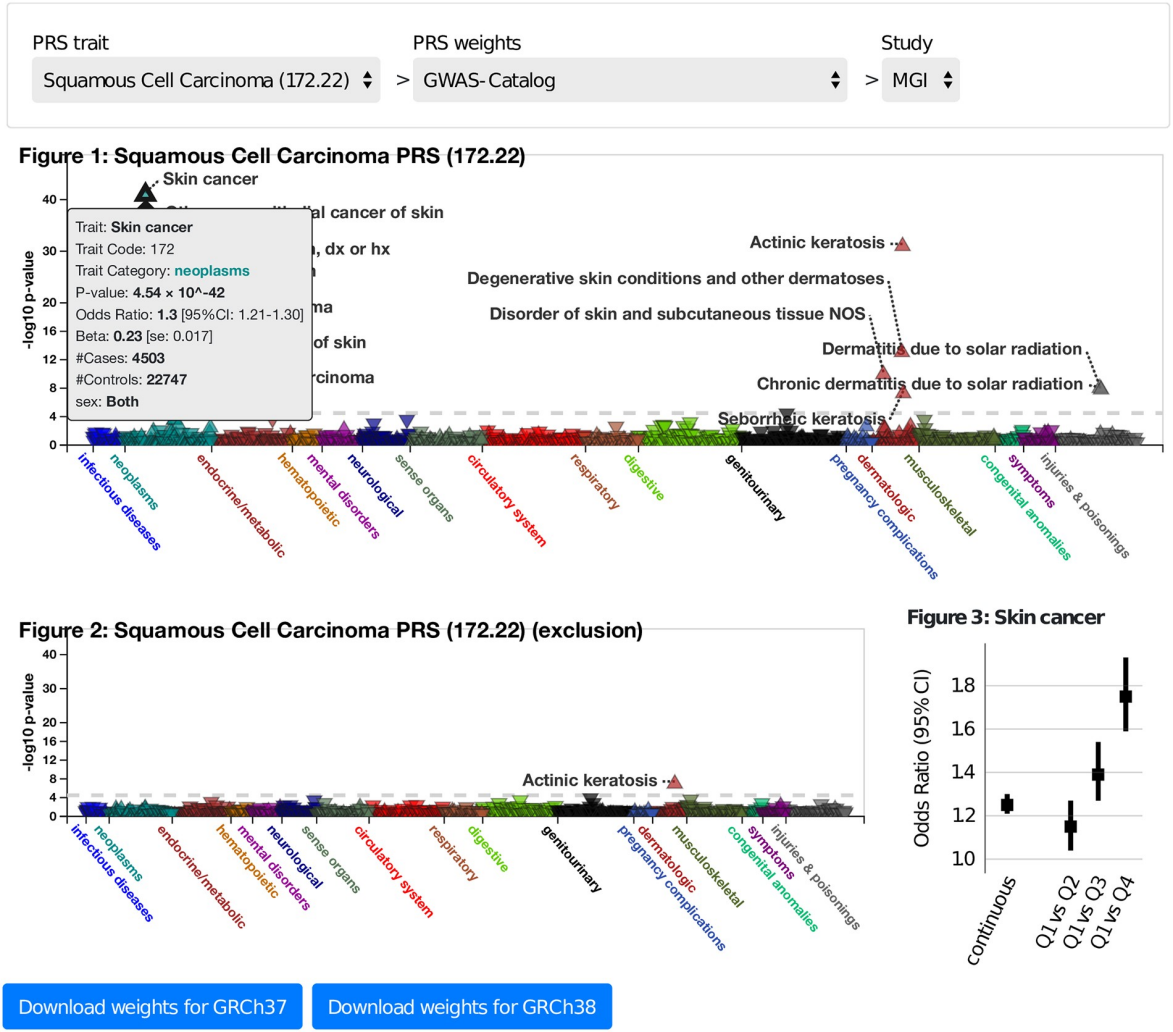
Example view from PRSweb (see web resources). A selection menu on top allows selection of PRS constructs and phenome while interactive plots with “PheWAS results” and “Exclusion PheWAS results” are generated after selection. “Associations between PRS and Selected Phenotype” plots are generated after clicking on a triangle in the PheWAS plots. Detailed summary statistics for each trait association are provided in mouseover elements (shown in grey). Underlying weights of a selected PRS can be downloaded via bottons below the plots (blue).

For comparison of the aforementioned PRS-PheWAS results and to provide researchers with resources for future PRS-based analyses, we developed an open access, online visual catalog *PRSweb* available at https://statgen.github.io/PRSweb that enables interactive exploration of the PheWAS results for each of the skin cancer subtypes and different PRS construction methods explored in this paper, for both the MGI and UK Biobank phenomes. *PRSweb* shows PRS-PheWAS plots with various choices of PRS in the drop-down menu (example screenshot in Fig 3) and offers downloadable PRS constructs (list of independent risk variants with corresponding weights). Mouse-over boxes offer detailed information about top results if needed, without impeding the overall user experience (grey box in Fig 3). Enrichment of cases in the upper quartiles of the PRS distribution are presented in forest plots.

## Discussion

Polygenic risk scores combine information from a large number of genetic variants to stratify subjects in terms of their risk of developing a particular disease. In the first aim of this paper, we demonstrate that PRS can also be used to explore shared and unique genetic risk profiles and secondary phenotype associations for related disease subtypes. We focus our attention on the setting of skin cancer, but the statistical approach can be applied to study other diseases with well-defined molecular subtypes.

For each skin cancer subtype, we constructed PRS using various PRS construction methods and evaluated their associations to the overall skin cancer phenotype and the three most common skin cancer subtypes: melanoma, basal cell carcinoma, and squamous cell carcinoma. We demonstrated that PRS constructed using EHR-derived phenotypes can sometimes (but not always) have good performance in terms of specificity for the primary phenotype. All PRS were positively associated with all skin cancer phenotypes under consideration. This suggests that each individual PRS may capture some shared genetic contributions for disease development across skin cancer subtypes.

For each skin cancer subtype, we performed a PRS-PheWAS to identify secondary phenotypes that are associated with the corresponding PRS. We identified many dermatological features in addition to the primary phenotype, indicating the ability of PRS to reproduce associations with the primary phenotype even after multiple testing corrections and covariate adjustment. The majority of these associations were replicated in a PRS-PheWAS performed for the UK Biobank phenome. Our analyses identified actinic keratosis, which is believed to be a precursor to squamous cell and basal cell carcinoma, as an independent predictor of basal cell and squamous cell carcinoma, and we demonstrated that incorporating the PRS in addition to clinical information improved discrimination for future skin cancer diagnoses (**Appendix 1 in** S1 Text) [46–48]. Additionally, some secondary phenotypes (for example, diseases of the sebaceous glands and sebaceous cysts) were identified in PRS-PheWAS only for the non-melanoma subtypes, which may provide some insight into some differentiating features of these subtypes.

In an additional analysis, we identified loci that were shared among all three skin cancer subtypes’ PRS. Loci overlap between the PRS for the three subtypes may indicate factors related to common biology between the subtypes. We noted that all shared loci (*HERC2*/*OCA2*, *IRF4*, *MC1R*, *RALY*, *SLC45A2* and *TYR*) were also loci that had been associated with human pigmentation traits and/or harbor key genes of the biochemical pathway of melanogenesis [49, 53–61, 63, 66–70]. To more directly explore secondary associations common to all skin cancers, we constructed PRS using SNPs shared by all three skin cancer subtypes and a PRS for skin tanning ability using results from a recent GWAS meta-analysis.[69] The skin tanning ability PRS PheWAS identified a very similar set of traits to the shared PRS PheWAS, suggesting that the shared genetic component may in part represent genetic factors influencing the skin pigmentation and the skin reaction to sun exposure. In an attempt to more directly identify secondary associations unique to each subtype, we also constructed PRS using SNPs unique to each subtype’s PRS. This analysis did not identify any strong subtype-specific associations, perhaps suggesting that the main genetic drivers of skin cancer are shared across subtypes.

In this paper, we explore strategies for constructing a PRS using markers and weights obtained from various publicly-available sources. We compare three general strategies for PRS construction. In the first strategy, we consider PRS constructed using a small number of markers and weights identified from either the latest GWAS or GWAS meta-analysis or the NHGRI-EBI GWAS catalog. We first compare these two PRS construction methods in terms of their associations with related and unrelated EHR-derived phenotypes. A priori, we may have some belief that the latest (and often the largest) GWAS may provide a better source of evidence to use for PRS construction due to larger sample sizes and (potentially) more carefully curated data. The latest GWAS and GWAS catalog methods produced PRS with similar performance in terms of their associations with and discrimination for the primary phenotype used to construct the PRS for both basal cell carcinoma and melanoma. The latest GWAS method produced better results for squamous cell carcinoma.

In the second PRS construction strategy, we use UK Biobank summary statistics at different p-value depths to construct PRS. We found that incorporating additional loci that did not reach genome-wide significance did improve the PRS’ ability to discriminate cases from controls for the primary phenotype up to a point. In particular, PRS constructed using SNPs with p-values less than 5x10^-8^ or 5x10^-7^ resulted in the best performance, but further increasing the p-value threshold resulted in reduced performance. Crucially, we also observed stronger associations between the PRS and secondary phenotypes for PRS constructed using depths of 5x10^-8^ and 5x10^-7^. These results suggest that some benefit may be observed by incorporating loci that do not reach significance into the PRS construction but incorporating too many loci with larger p-values may not improve the predictive ability of the PRS (for both primary and secondary phenotypes). However, this gain or reduction in PRS performance may depend on the phenotype of interest and on the prevalence of the phenotype in the analytical sample.

In the third PRS construction strategy, we use the LDpred method to construct PRS using the whole spectrum of observed genetic information. For melanoma, the LDpred PRS which modelled smaller fractions of causal variants were favored over the ones modelling larger fractions. While the PRS construction with LDpred performed similarly to the various depth approach in our particular study, its computational cost was substantially higher. However, recent work indicated that LDpred might outperform pruning and thresholding approaches when larger training data sets are available [71].

In our study, PRS associations with the primary phenotype were generally stronger using the latest GWAS and GWAS catalog-based PRS than for the PRS constructed using LDpred or using UK Biobank summary statistics at different depths. Since the underlying case numbers in the discovery GWAS for the former were substantially larger than the case numbers in the UK Biobank GWAS, no direct comparison between approaches was possible, and also because the required full summary statistics of the contributing and larger skin cancer case-control studies of the latest GWAS and GWAS catalog entries were not made available. Additionally, these simpler PRS construction strategies appeared to more clearly differentiate between related subtypes than the genome-wide PRS construction methods. All three PRS construction strategies produced many similar secondary associations from PRS-PheWAS, as shown in Table 3. Overall, these simpler PRS construction methods worked well in our particular skin cancer setting, and we did not see any improvements to using a much larger number of SNPs in the PRS construction. Future releases of full summary statistics from large skin cancer GWAS meta-analyses will enable more liberal thresholds and consequently may result in better performing PRS [3, 72, 73].

As a product of this study, we provide an online visual catalog *PRSweb* (see **Web resources**) that provides PRS-PheWAS results for the various skin cancer phenotypes for PRS constructed using the different methods explored in this paper. *PRSweb* will provide a routine way to compare different PRS construction methods and to explore PRS-PheWAS results in detail. Additionally, *PRSweb* provides the PRS construction details, which researchers can download and use in their own analyses. In the future, we plan to extend this online platform to include PheWAS for many other cancer phenotypes, which will make this online platform a general tool for identifying phenotypes related to particular types of cancer.

One limitation of the generalizability of this study comes from the homogeneous race profile of MGI and UK Biobank. UK Biobank consists of subjects of primarily European descent, and we restricted our analyses to subjects of European descent in MGI (excluding about 10% of the subjects in MGI) in order to ensure greater comparability between the two datasets. Additionally, many of the existing GWAS were conducted on European populations, and we wanted to consider similar samples when comparing the performance of PRS constructed using summary statistics from European populations. Unlike UK Biobank, MGI is not a population-based sample; rather, it is a sample of patients recruited from a large academic medical center. Patients were recruited prior to surgery through the anesthesiology department, and therefore they may present a potential for selection bias. PRS-PheWAS results are susceptible to collider bias caused by PRS relationships with both skin cancer diagnosis and other diseases related to sampling. The exclusion PheWAS strategy attempts to overcome this obstacle by evaluating PRS associations with secondary phenotypes only in the subjects without a skin cancer diagnosis. This approach does not remove the possibility of sampling bias, but it may help reduce the impact of sampling on the estimated PRS-phenotype associations. The chosen design of matched case-control studies can reduce bias and detection of false positives compared to the unmatched analysis, but it is typically less powerful than the unmatched analysis (**Fig N in** S1 Text).

An additional limitation for all EHR-based phenome-wide studies is the potential for bias due to phenotype misclassification. In **S1 Section 1**, we discuss this issue in more detail, and we provide a sensitivity analysis exploring the impact of misclassification on study results in **Fig O in** S1 Text. In spite of these limitations, a principled comparison of the various methods explored in this paper may provide researchers with a sense of the robustness of their PheWAS inference to the PRS construction method and an analytical framework for the exploration of shared genetic architecture of related traits.

### Web resources

PRSweb; https://statgen.github.io/PRSweb University of Michigan Medical School Central Biorepository; https://research.medicine.umich.edu/our-units/central-biorepository UK Biobank; http://www.ukbiobank.ac.uk/ UK Biobank GWAS summary statistics; https://tinyurl.com/UKB-SAIGE TOPMed variant browser, https://bravo.sph.umich.edu/freeze5/hg38/ TOPMed program, https://www.nhlbi.nih.gov/science/trans-omics-precision-medicine-topmed-program Minimac4; https://genome.sph.umich.edu/wiki/Minimac4 BCFtools; https://samtools.github.io/bcftools/bcftools.html KING; http://people.virginia.edu/~wc9c/KING/ FASTINDEP; https://github.com/endrebak/fastindep PLINK; https://www.cog-genomics.org/plink2/ Eagle; https://data.broadinstitute.org/alkesgroup/Eagle/ UCSC Genome Browser; http://genome.ucsc.edu/ R; https://cran.r-project.org/ NHGRI-EBI GWAS Catalog; https://www.ebi.ac.uk/gwas/ dbSNP; https://www.ncbi.nlm.nih.gov/projects/SNP/ Imputation server; https://imputationserver.sph.umich.edu/ Jinja, https://github.com/pallets/jinja Locuszoom, https://github.com/statgen/locuszoom.

## Supporting information

S1 Text Supporting material. This file contains supporting Appendices 1–2, Figures A-O and Tables A-E.
(PDF)

S1 File Supporting tables. This Excel file contains the following tables: Sheet 1: Table F, ICD9 codes to PheWAS code mapping tables for skin cancer; Sheet 2: Table G, ICD10 codes to PheWAS code mapping tables for skin cancer; Sheet 3: Table H, MGI and UK Biobank phenome summary; Sheet 4: Table I, GWAS Catalog and latest GWAS risk SNP selection; Sheet 5: Table J, UKB GWAS risk SNP selection; Sheet 6: Table K, Omnibus table of significant results.
(XLSX)
