# Supplementary material for: Exploring various polygenic risk scores for skin cancer in the phenomes of the Michigan genomics initiative and the UK Biobank with a visual catalog: PRSWeb
Source: PLoS Genet. 2019 Jun 13;15(6):e1008202. doi: 10.1371/journal.pgen.1008202 (PMC6592565; doi:10.1371/journal.pgen.1008202)
Supplement: S1 Text — This file contains supporting Appendices 1–2, Figures A-O and Tables A-E. (PDF) [file pgen.1008202.s001.pdf]

Supplement to "Exploring Various Polygenic Risk Scores for Skin Cancer in the Phenomes of the Michigan Genomics Initiative and the UK Biobank with a Visual Catalog: *PRSWeb*"

Appendix 1: Sub-analysis of actinic keratosis as a predictor of future skin cancer

Actinic keratosis (AK) is a rough, scaly patch of skin that usually develops after years of cumulative skin exposure [1]. Previous research has identified actinic keratosis as a common pre-malignant condition for squamous cell carcinoma (SCC) [2]. Actinic keratosis has also been identified as a potential precursor to basal cell carcinoma (BCC) [3, 4]. The availability of temporal information of diagnoses in the MGI cohort offered the opportunity to explore actinic keratosis as a potential precursor for development of skin cancer in MGI.

Fig E in Text S1 shows the ROC curves and AUC values for diagnosis of actinic keratosis at least one year before any skin cancer diagnosis and its association with future BCC or SCC diagnosis. AK diagnosis alone has little discrimination abilities, with AUC values of 0.52 (95% CI [0.51, 0.53]) for BCC and 0.51 (95% CI [0.50, 0.52]) for SCC. The bPRS and sPRS provide comparatively good discrimination (AUC 0.64 [0.62, 0.65] for BCC and 0.59 [0.57, 0.61] for SCC). The combination of prior AK diagnosis and bPRS provided further improvement in discrimination, with an AUC of 0.65 (95% CI [0.64, 0.67]).

Tables A and B in Text S1 provides odds ratio estimates relating AK and the PRS to future BCC and SCC diagnosis. The odds of BCC diagnosis were significantly higher in subjects with a prior actinic keratosis diagnosis (OR 1.46, 95% CI [1.18, 1.80]). Notably, when we adjust for both bPRS and AK diagnosis, the unadjusted and adjusted effects of both variables are similar, suggesting that AK diagnosis may be an independent predictor of future BCC diagnosis. In contrast, AK diagnosis was *not* an independent predictor of SCC diagnosis. **Fig F in Text S1** shows the timing of an AK diagnosis relative to a skin cancer diagnosis for patients with both diagnoses. For subjects with basal cell carcinoma or squamous cell carcinoma, AK diagnoses tended to occur prior to the skin cancer diagnosis (often within 8 years).

Appendix 2: Phenotype Misclassification

An important issue facing EHR-based research is the definition of disease phenotypes. It is well-known that phenotypes based on ICD codes (or corresponding aggregate codes called “phecodes”) have a large potential to be misclassified. Statistical methods have been developed to incorporate a larger amount of EHR information to help improve the accuracy of the phenotype assignments, but these methods are inherently limited by the information available in the EHR.

A natural question, then, is the impact of phenotype misclassification on the results of association analyses like those performed in this paper. In a recent paper, we developed statistical methods for accounting for phenotype misclassification on inference under a standard logistic regression model [5]. Slightly extending the results in that paper to incorporate differential bi-directional phenotype misclassification, we can express the association parameter we estimate from fitting the model to misclassified data as a function of the true sensitivity and specificity of the phenotype in our data.

Since we expect the specificity (the proportion of subjects without the disease to be listed as non-diseased) to be fairly high, e.g. greater than 0.8, we expect phenotype misclassification to generally result in bias in PRS-PheWAS association parameters toward the null. Therefore, we expect our false-positive rate and power to be lower than if we did not have phenotype misclassification. Findings that are phenome-wide significant are not expected to be the result of bias away from the null caused by phenotype misclassification.

As a sensitivity analysis, we explored how our estimated association between the BCC PRS and actinic keratosis diagnosis might be impacted by imperfect sensitivity and specificity for diagnosing actinic keratosis (**Figure M in Text S1**). For actinic keratosis, we believe specificity should be fairly high, but sensitivity may plausibly be somewhat low. This figure demonstrates that the expected actinic keratosis-BCC PRS association is not “explained away” by accounting for misclassification of the actinic keratosis phenotype.

Supplement References

1. Ko CJ. Actinic keratosis: facts and controversies. Clin Dermatol. 2010;28(3):249-53. Epub 2010/06/15. doi: 10.1016/j.clindermatol.2009.06.009. PubMed PMID: 20541675.
2. Fuchs A, Marmur E. The kinetics of skin cancer: progression of actinic keratosis to squamous cell carcinoma. Dermatol Surg. 2007;33(9):1099-101. Epub 2007/09/01. doi: 10.1111/j.1524-4725.2007.33224.x. PubMed PMID: 17760601.
3. Cohen JL. Actinic keratosis treatment as a key component of preventive strategies for nonmelanoma skin cancer. J Clin Aesthet Dermatol. 2010;3(6):39-44. Epub 2010/08/21. PubMed PMID: 20725550; PubMed Central PMCID: PMCPMC2921751.
4. Jacobs RJ, Phillips G. Basal cell carcinoma mistaken for actinic keratosis. Clin Exp Optom. 2006;89(3):171-5. Epub 2006/04/28. doi: 10.1111/j.1444-0938.2006.00032.x. PubMed PMID: 16637973.
5. Beesley LJ, Fritsche LG, Mukherjee B. A Modeling Framework for Exploring Sampling and Observation Process Biases in Genome and Phenome-wide Association Studies using Electronic Health Records. 2018:499392. doi: 10.1101/499392 %J bioRxiv.
6. Welch JW. Carcinoma arising in Sebaceous cysts. AMA Arch Surg. 1958;76(1):128-32. Epub 1958/01/01. PubMed PMID: 13487060.

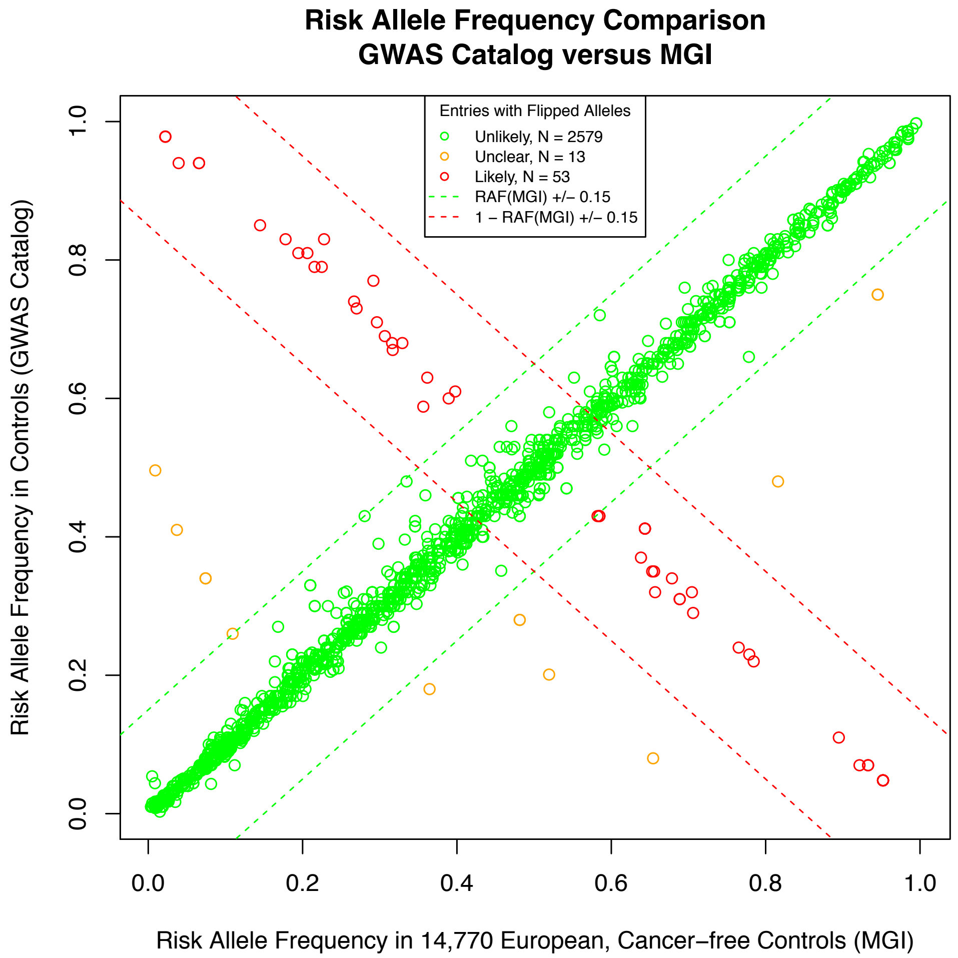

Fig A. Comparison of the Risk Allele Frequencies in the GWAS Catalog vs. MGI

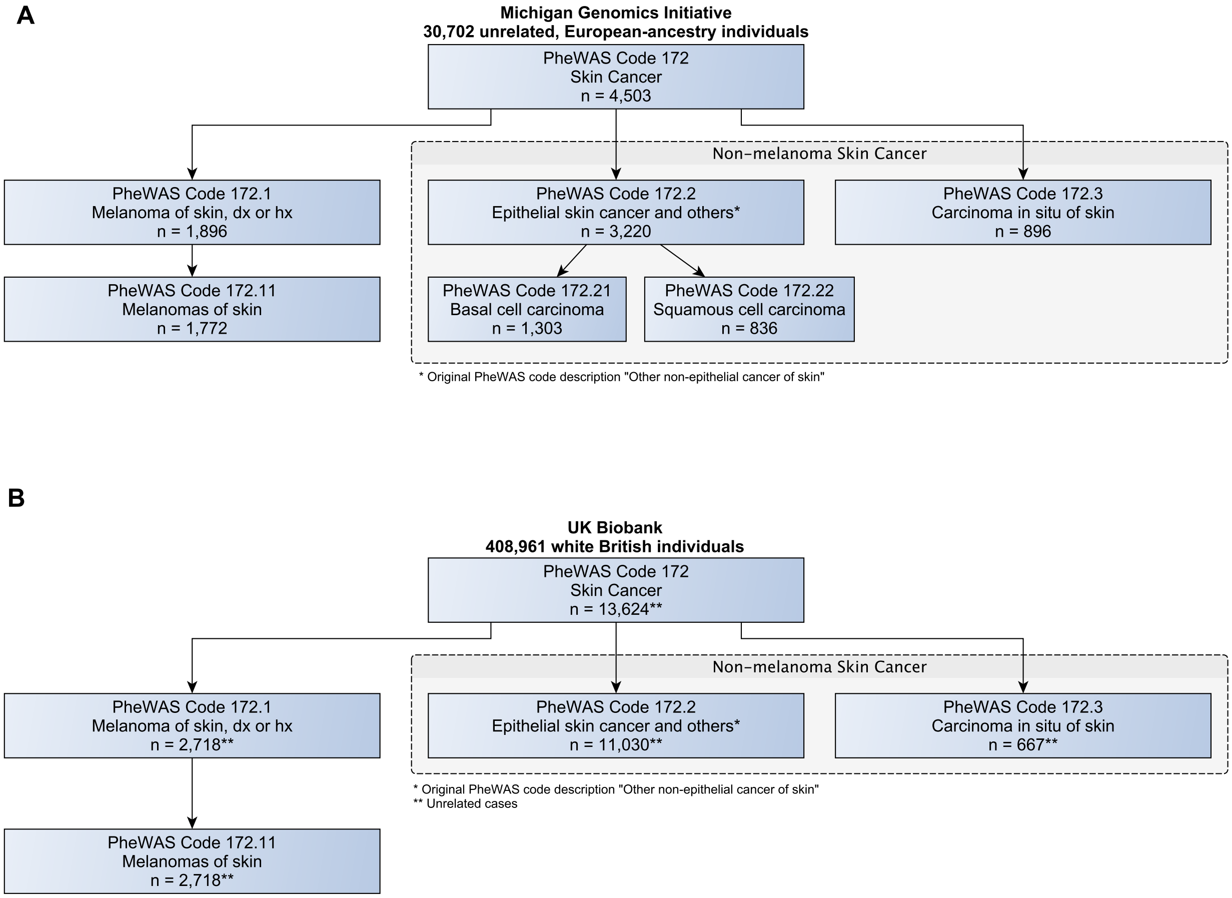

Fig B. Skin cancer PheWAS codes in (**A**) MGI and (**B**) UK Biobank

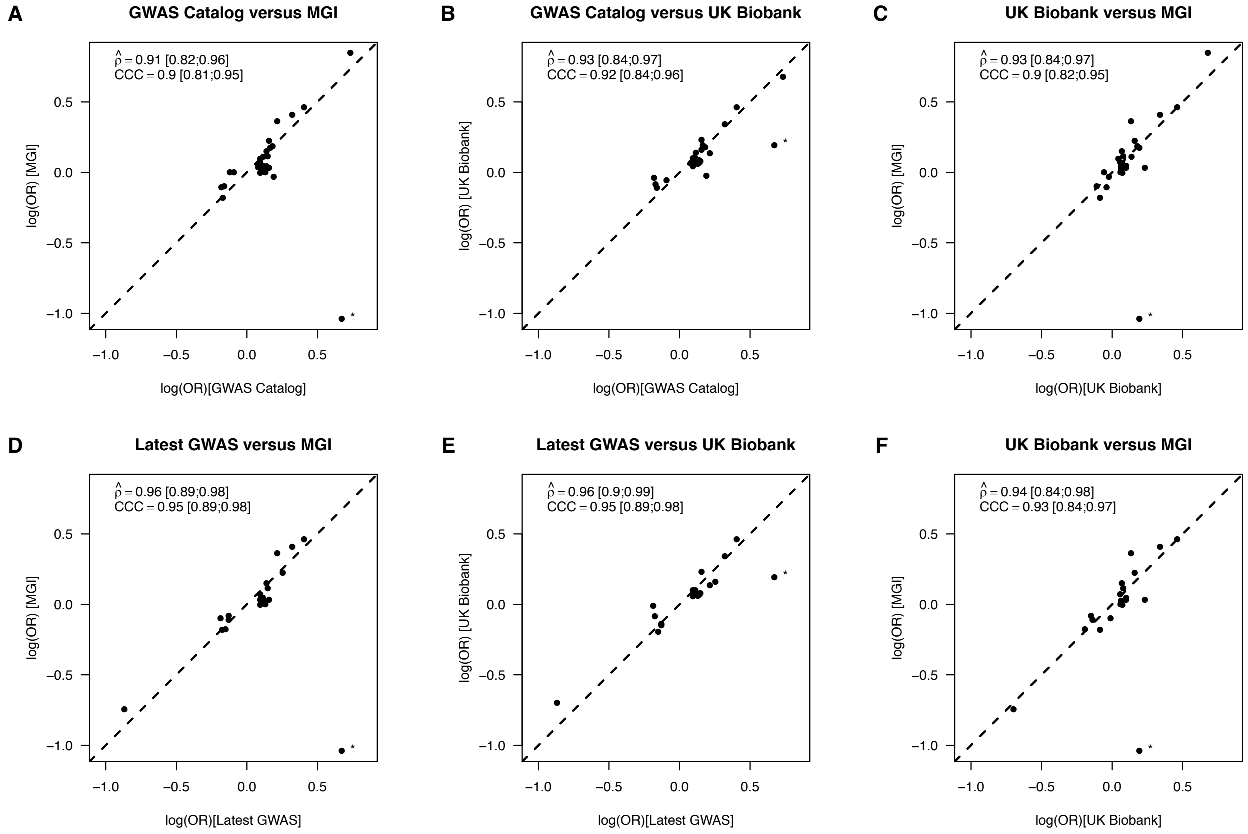

Fig C. A Comparison of the Melanoma PRS Construction Weights to SNP Associations Observed in MGI and UK Biobank. **(A) – (C)** correspond to SNPs included in the GWAS catalog PRS for Melanoma, and **(D) – (F)** correspond to SNPS included in the latest GWAS PRS for Melanoma. * This point is a clear outlier. This SNP was not used to estimate the correlation ($\hat{\rho}$) or the concordance measure (CCC).

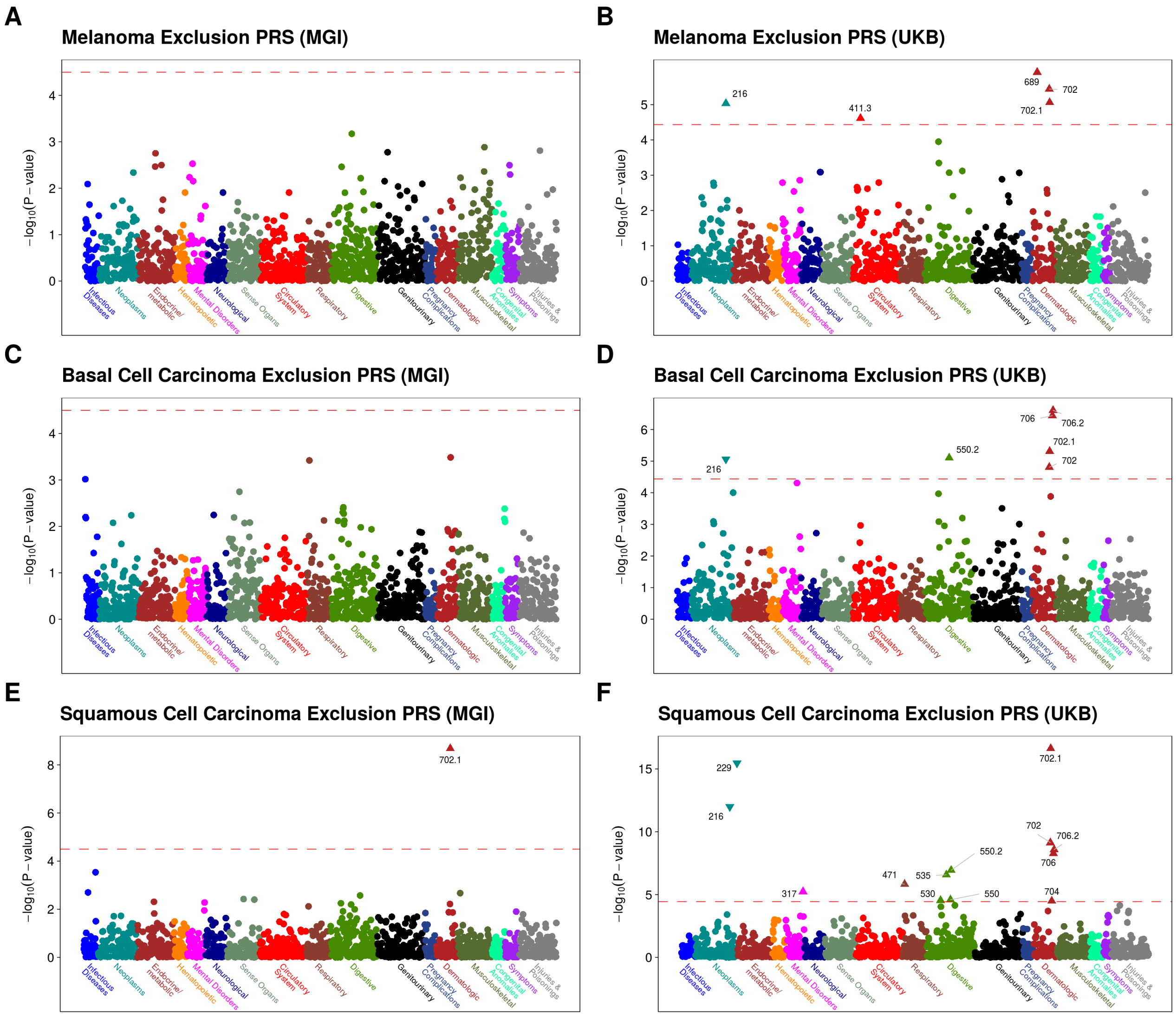

PheCode	Description	PheCode	Description	PheCode	Description	PheCode	Description
216	Benign neoplasm of skin	471	Nasal polyps	550.2	Diaphragmatic hernia	704	Diseases of hair and hair follicles
229	Benign neoplasm of unspecified sites	530	Diseases of esophagus	689	Disorder of skin and subcutaneous tissue NOS	706	Diseases of sebaceous glands
317	Alcohol-related disorders	535	Gastritis and duodenitis	702	Degenerative skin conditions and other dermatoses	706.2	Sebaceous cyst
411.3	Angina pectoris	550	Abdominal hernia	702.1	Actinic keratosis		

Fig D. Exclusion PRS PheWAS of the MGI and UKB phenomes*
The horizontal line indicates phenome-wide significance.
*These analyses exclude subjects with any skin cancer diagnosis

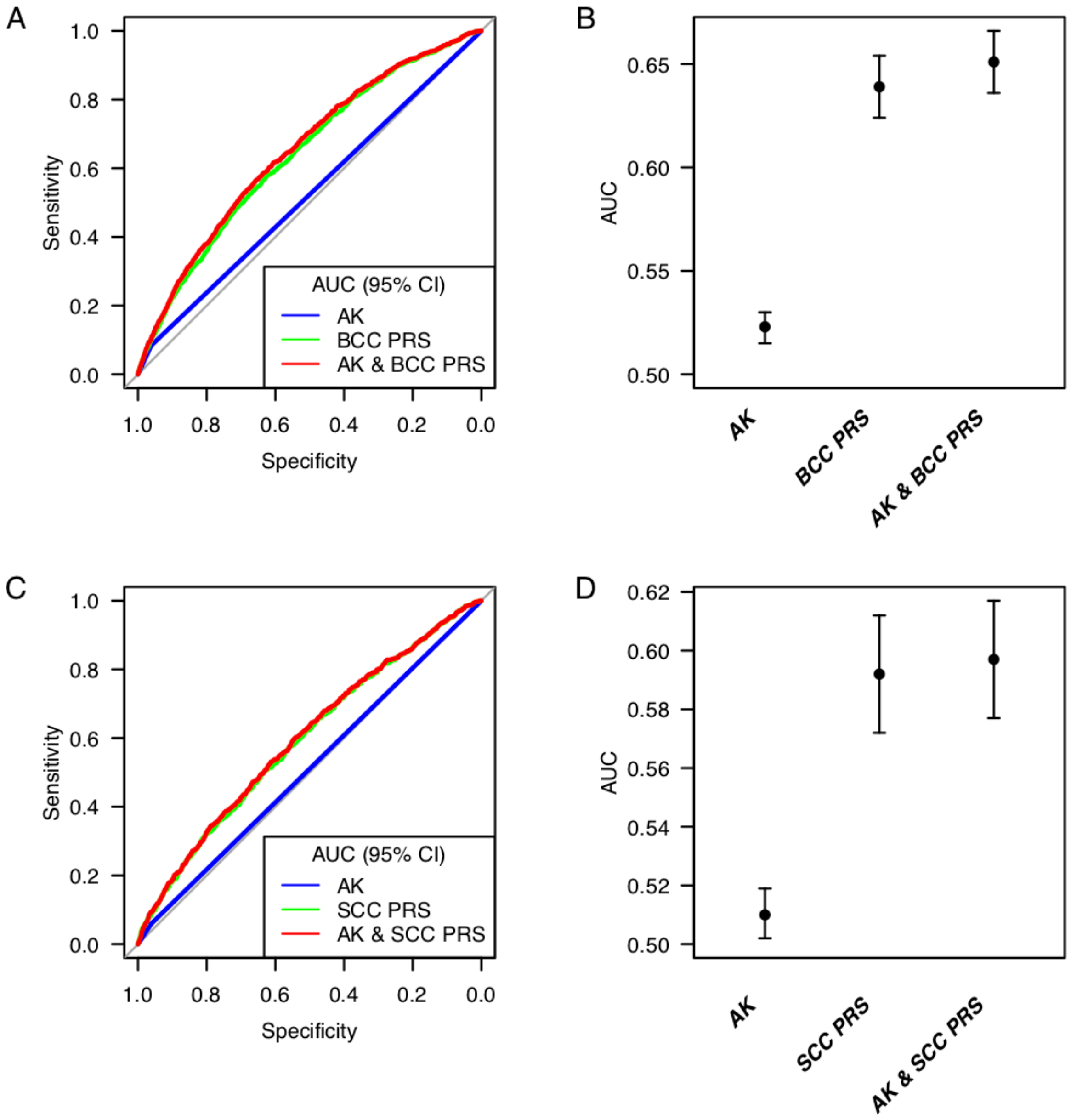

Fig E Comparison of predictors. Actinic keratosis (AK), at least 365 prior to any skin cancer diagnosis as predictor for basal cell carcinoma (BCC) (A and B) and squamous cell carcinoma (SCC) (C and D). The PRS for BCC and SCC as well as the combined predictors are shown for comparison.

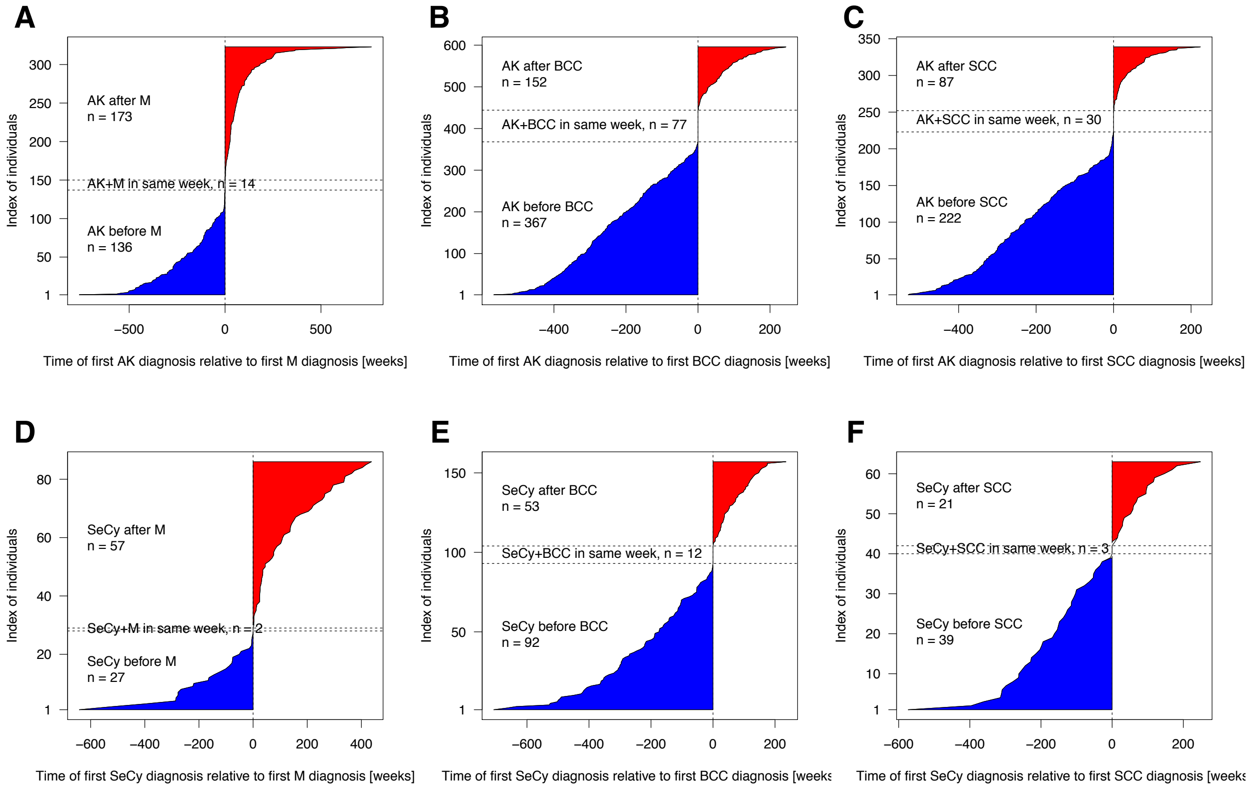

Fig F. Temporal order of diagnoses: (**A**) actinic keratosis (AK) and melanoma (M) in 323 individuals with AK and melanoma; (**B**) actinic keratosis (AK) and basal cell carcinoma (BCC) in 596 individuals with AK and BCC; (**C**) actinic keratosis (AK) and squamous cell carcinoma (SCC) in 339 individuals with AK and SCC; (**D**) Sebaceous cyst (SeCy) and melanoma (M) in 86 individuals with SeCy and melanoma; (**E**) Sebaceous cyst (SeCy) and basal cell carcinoma (BCC) in 157 individuals with SeCy and BCC; and (**F**) Sebaceous cyst (SeCy) and squamous cell carcinoma (SCC) in 63 individuals with SeCy and SCC. The time of the first non-cancer diagnosis relative to the cancer diagnosis is shown in weeks; before (blue) and after (red) the cancer diagnosis. Note: Since the phenotype "sebaceous cyst" and its over-category "Diseases of the sebaceous gland" remained significant in the UK Biobank exclusion PheWAS for basal cell and squamous cell carcinoma, we performed an exploration of potential temporal associations between diagnosis of sebaceous cyst and skin cancer. Sebaceous cysts are lumps under the skin often caused by blocked glands or swollen hair follicles in the skin and are often the result of minor irritation or injury. Rarely, these cysts have been known to undergo malignant transformation into non-epithelial carcinoma.[6] Although not shown, prior diagnosis of sebaceous cyst was not an independent predictor of the development of SCC or BCC, indicating that the association between the PRS and the sebaceous cyst phenotype may be driven by shared genetic risk with BCC and SCC

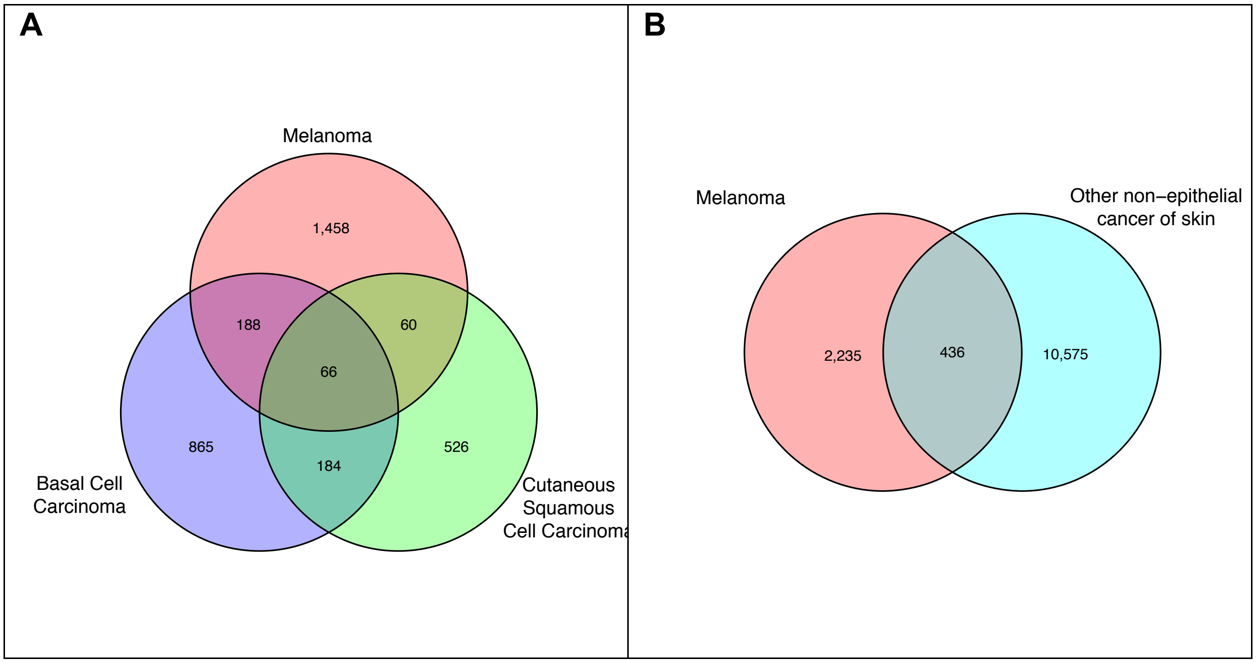

Fig G. Number of subjects with various skin cancer phecodes in MGI (**A**) and the UK Biobank (**B**).

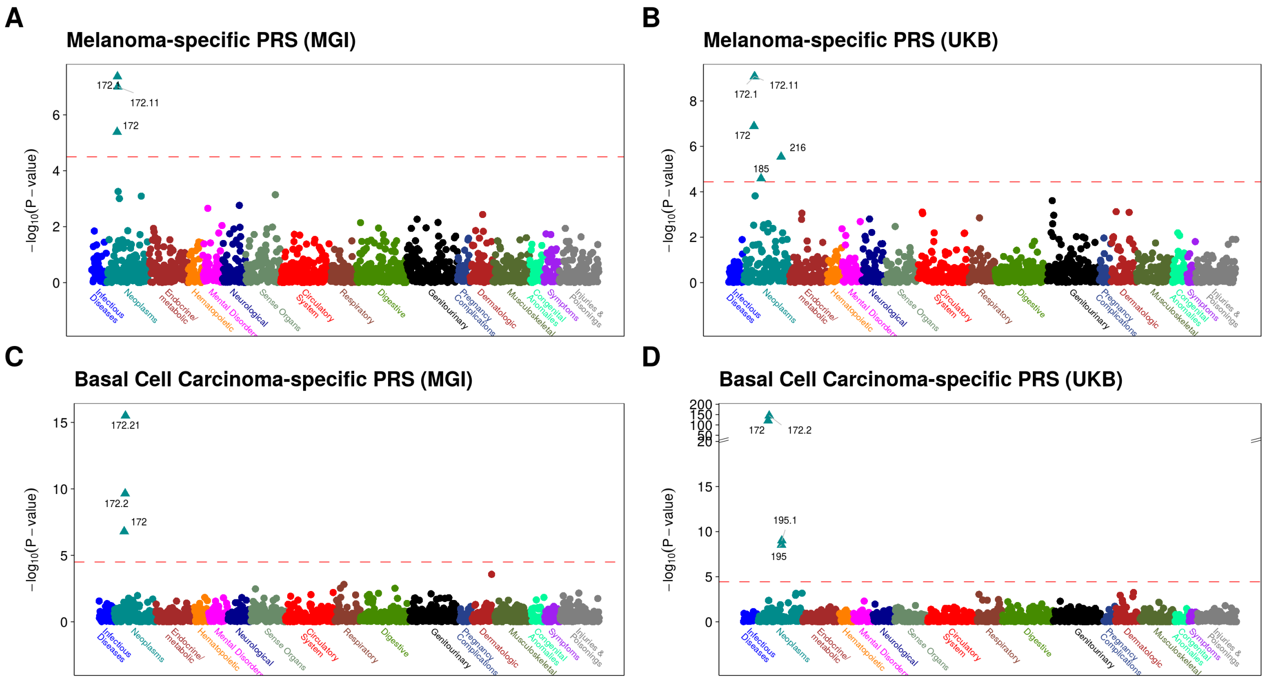

PheCode	Description	PheCode	Description	PheCode	Description
172	Skin cancer	172.2	Other non-epithelial cancer of skin	195	Cancer, suspected or other
172.1	Melanomas of skin, dx or hx	172.21	Basal cell carcinoma	195.1	Malignant neoplasm, other
172.11	Melanomas of skin	185	Cancer of prostate	216	Benign neoplasm of skin

Fig H. PheWAS of subtype-specific PRS in MGI (A&C) and in UK Biobank (B&D). The horizontal line indicates phenome-wide significance.

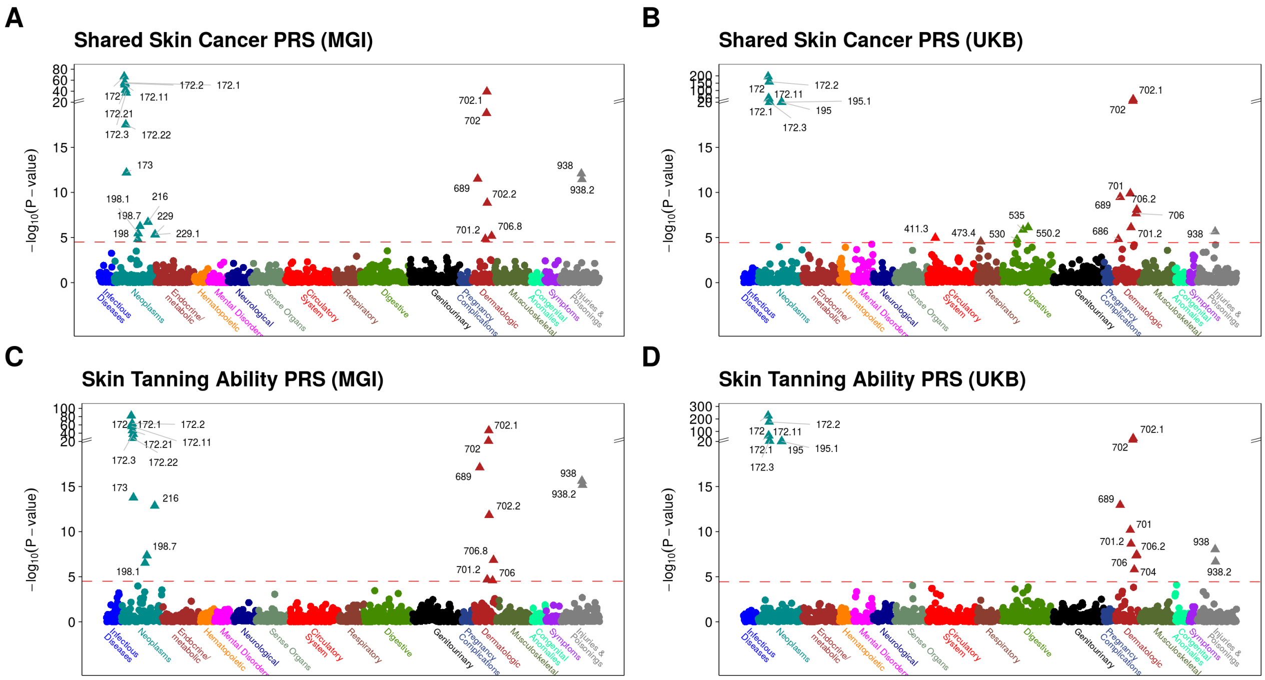

PheCode	Description	PheCode	Description	PheCode	Description	PheCode	Description
172	Skin cancer	195.1	Malignant neoplasm, other	530	Diseases of esophagus	702.2	Seborrheic keratosis
172.1	Melanomas of skin, dx or hx	198	Secondary malignant neoplasm	535	Gastritis and duodenitis	704	Diseases of hair and hair follicles
172.11	Melanomas of skin	198.1	Secondary malignancy of lymph nodes	550.2	Diaphragmatic hernia	706	Diseases of sebaceous glands
172.2	Other non-epithelial cancer of skin	198.7	Secondary malignant neoplasm of skin	686	Other local infections of skin and subcutaneous tissue	706.2	Sebaceous cyst
172.21	Basal cell carcinoma	216	Benign neoplasm of skin	689	Disorder of skin and subcutaneous tissue NOS	706.8	Other specified diseases of sebaceous glands
172.22	Squamous cell carcinoma	229	Benign neoplasm of unspecified sites	701	Other hypertrophic and atrophic conditions of skin	938	Dermatitis due to solar radiation
172.3	Carcinoma in situ of skin	229.1	Benign neoplasm of lymph nodes	701.2	Scar conditions and fibrosis of skin	938.2	Chronic dermatitis due to solar radiation
173	Neoplasm of uncertain behavior of skin	411.3	Angina pectoris	702	Degenerative skin conditions and other dermatoses		
195	Cancer, suspected or other	473.4	Voice disturbance	702.1	Actinic keratosis		

Fig I. PheWAS on shared skin cancer risk PRS and skin saturation PRS. The horizontal line indicates phenome-wide significance. The shared skin cancer PRS represents the unweighted sum of risk allele counts of seven independent SNPs of six risk loci that are shared between melanoma, basal cell carcinoma, and squamous cell carcinoma. The skin tanning ability PRS consists of 26 uncorrelated SNPs previously found to be associated with the skin's ability to suntan. The two PRS have six overlapping loci/SNPs (**Fig 2**).

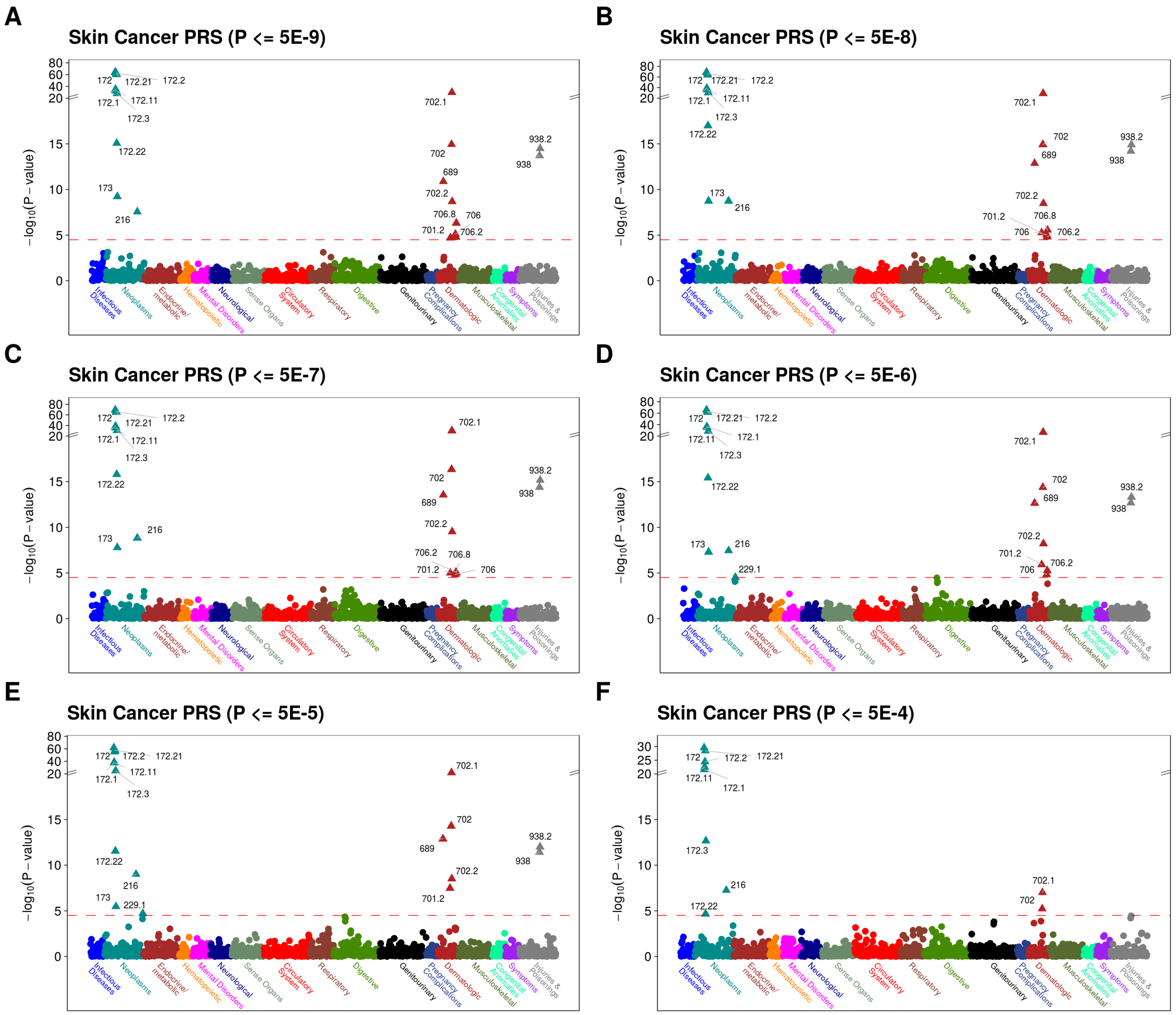

PheCode	Description	PheCode	Description	PheCode	Description	PheCode	Description
172	Skin cancer	172.22	Squamous cell carcinoma	689	Disorder of skin and subcutaneous tissue NOS	706	Diseases of sebaceous glands
172.1	Melanomas of skin, dx or hx	172.3	Carcinoma in situ of skin	701.2	Scar conditions and fibrosis of skin	706.2	Sebaceous cyst
172.11	Melanomas of skin	173	Neoplasm of uncertain behavior of skin	702	Degenerative skin conditions and other dermatoses	706.8	Other specified diseases of sebaceous glands
172.2	Other non-epithelial cancer of skin	216	Benign neoplasm of skin	702.1	Actinic keratosis	938	Dermatitis due to solar radiation
172.21	Basal cell carcinoma	229.1	Benign neoplasm of lymph nodes	702.2	Seborrheic keratosis	938.2	Chronic dermatitis due to solar radiation

Fig J. PheWAS on Overall Skin Cancer and Melanoma PRS Constructed using UK Biobank Statistics at Different Depths. Results (**A-F**) are shown with increasing depth from left to right: P <= $5x10^{-9}$, $5x10^{-8}$, $5x10^{-7}$, $5x10^{-6}$, $5x10^{-5}$, $5x10^{-4}$

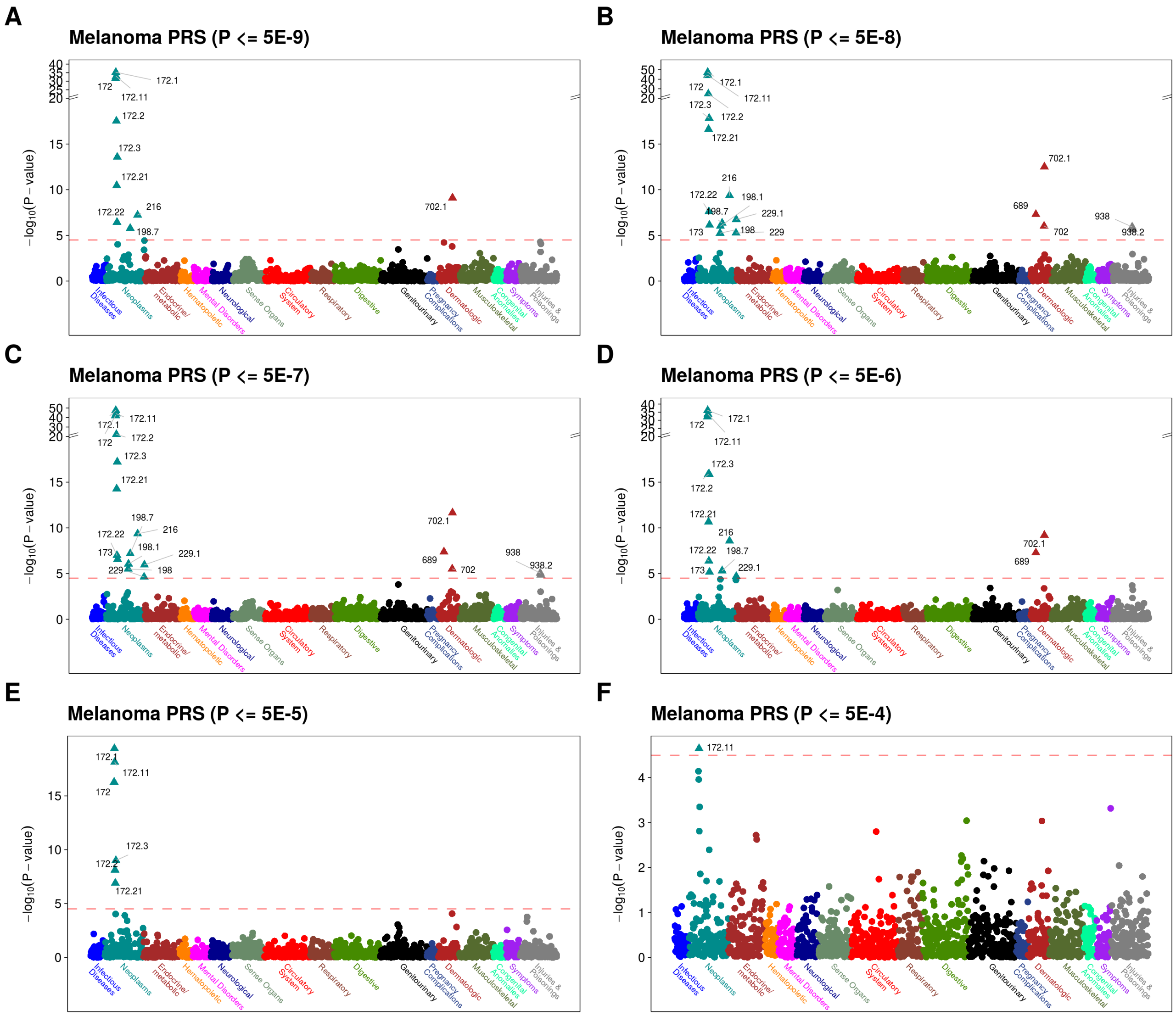

PheCode	Description	PheCode	Description	PheCode	Description	PheCode	Description
172	Skin cancer	172.22	Squamous cell carcinoma	198.7	Secondary malignant neoplasm of skin	702	Degenerative skin conditions and other dermatoses
172.1	Melanomas of skin, dx or hx	172.3	Carcinoma in situ of skin	216	Benign neoplasm of skin	702.1	Actinic keratosis
172.11	Melanomas of skin	173	Neoplasm of uncertain behavior of skin	229	Benign neoplasm of unspecified sites	938	Dermatitis due to solar radiation
172.2	Other non-epithelial cancer of skin	198	Secondary malignant neoplasm	229.1	Benign neoplasm of lymph nodes	938.2	Chronic dermatitis due to solar radiation
172.21	Basal cell carcinoma	198.1	Secondary malignancy of lymph nodes	689	Disorder of skin and subcutaneous tissue NOS		

Fig K. PheWAS on melanoma PRS constructed using UK Biobank statistics at different depths. Results are shown with increasing depth from (A – F): P <= $5x10^{-9}$, $5x10^{-8}$, $5x10^{-7}$, $5x10^{-6}$, $5x10^{-5}$, $5x10^{-4}$.

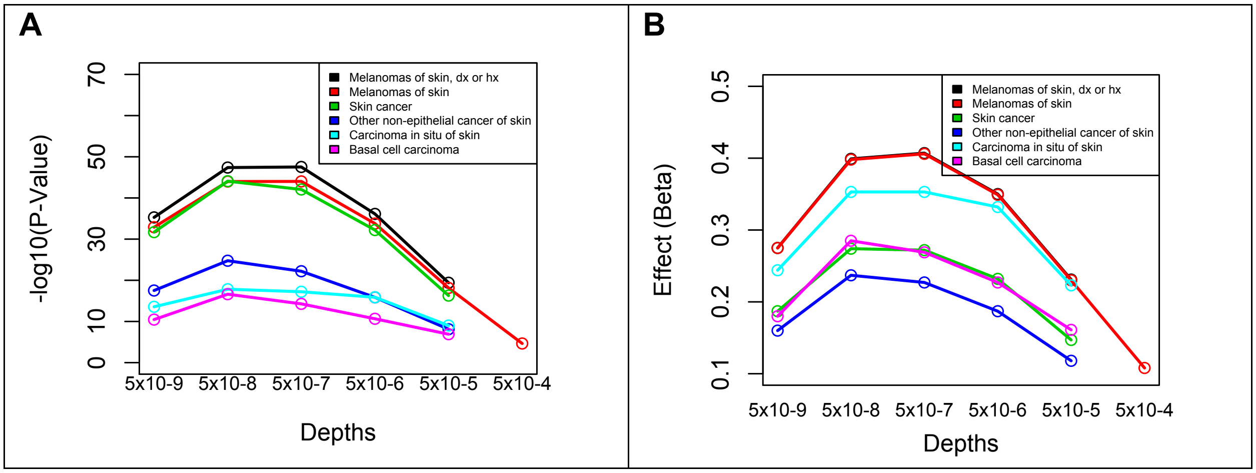

Fig L. (A) P-Values and **(B)** Effect Sizes/Betas for Most Significant Phenotypes Identified in MGI using PRS Constructed from UKB Summary Statistics at Different Depths

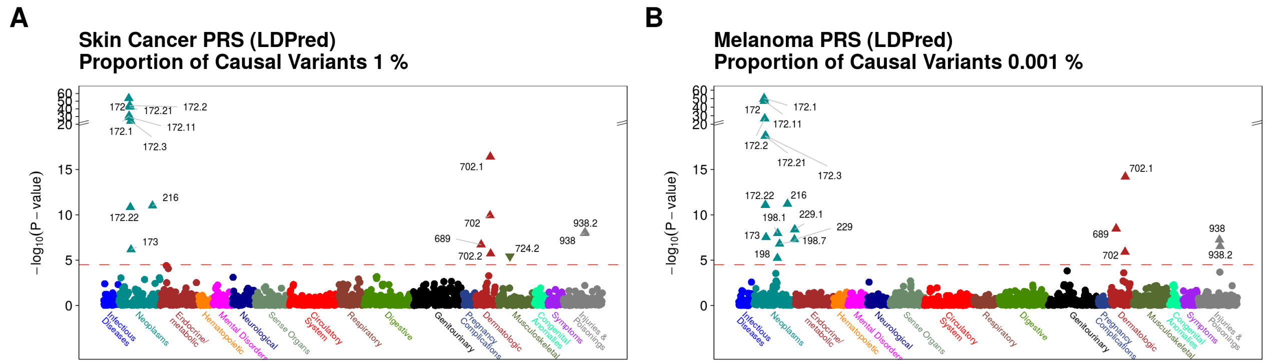

PheCode	Description	PheCode	Description	PheCode	Description	PheCode	Description
172	Skin cancer	172.3	Carcinoma in situ of skin	229	Benign neoplasm of unspecified sites	724.2	Disorders of coccyx
172.1	Melanomas of skin, dx or hx	173	Neoplasm of uncertain behavior of skin	229.1	Benign neoplasm of lymph nodes	938	Dermatitis due to solar radiation
172.11	Melanomas of skin	198	Secondary malignant neoplasm	689	Disorder of skin and subcutaneous tissue NOS	938.2	Chronic dermatitis due to solar radiation
172.2	Other non-epithelial cancer of skin	198.1	Secondary malignancy of lymph nodes	702	Degenerative skin conditions and other dermatoses		
172.21	Basal cell carcinoma	198.7	Secondary malignant neoplasm of skin	702.1	Actinic keratosis		
172.22	Squamous cell carcinoma	216	Benign neoplasm of skin	702.2	Seborrheic keratosis		

Fig M. PheWAS on Skin Cancer and Melanoma PRS constructed using LDpred. Results (**A-B**) correspond to the largest value for pseudo R^2 from **Table E in Text S1**.

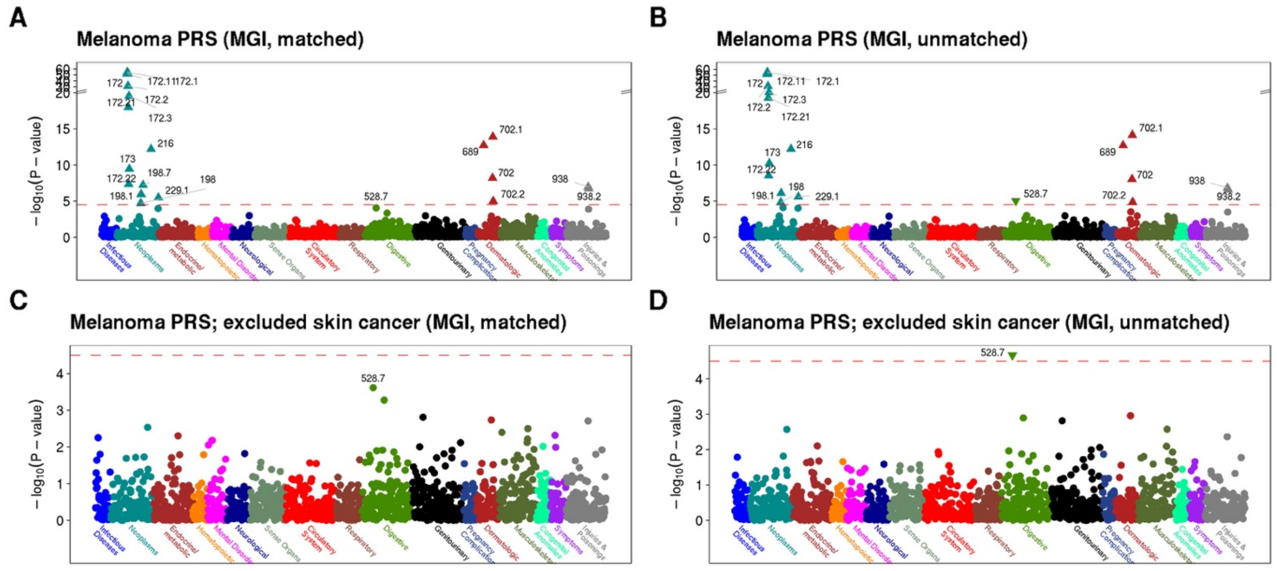

PheCode	Description	PheCode	Description	PheCode	Description	PheCode	Description
172	Skin cancer	172.22	Squamous cell carcinoma	198.7	Secondary malignant neoplasm of skin	702	Degenerative skin conditions and other dermatoses
172.1	Melanomas of skin, dx or hx	172.3	Carcinoma in situ of skin	216	Benign neoplasm of skin	702.1	Actinic keratosis
172.11	Melanomas of skin	173	Neoplasm of uncertain behavior of skin	229.1	Benign neoplasm of lymph nodes	702.2	Seborrheic keratosis
172.2	Other non-epithelial cancer of skin	198	Secondary malignant neoplasm	528.7	Sialolithiasis	938	Dermatitis due to solar radiation
172.21	Basal cell carcinoma	198.1	Secondary malignancy of lymph nodes	689	Disorder of skin and subcutaneous tissue NOS	938.2	Chronic dermatitis due to solar radiation

Fig N: PRS-PheWAS (A,B) and Exclusion PRS-PheWAS (C,D) in the matched (left) and unmatched (right) MGI phenome.

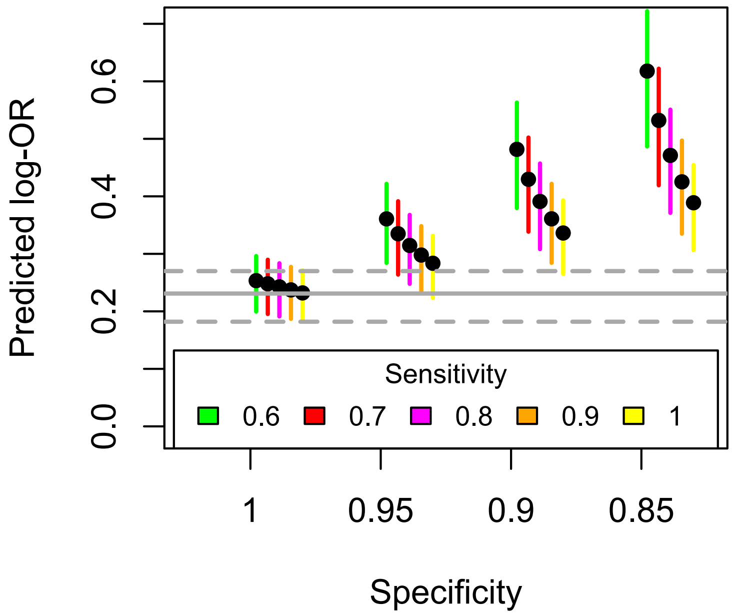

Fig O: Predicted log-OR between bPRS and actinic keratosis diagnosis accounting for different degrees of actinic keratosis diagnosis misclassification in MGI. Gray lines indicate our estimated log-OR and corresponding confidence intervals in MGI.

	OR[a] adjusting for PRS *or* AK (95% CI)	OR[a] adjusting for PRS *and* AK (95% CI)
BCC PRS	1.67 (1.58,1.77)	1.67 (1.57,1.77)
AK 365 days before any skin cancer diagnosis	1.46 (1.18,1.80)	1.41 (1.13,1.74)

Table A. Predictors for basal cell carcinoma (BCC) in MGI.
[a] Adjusting for Age, gender, array, and PCs 1-4

	OR[a] adjusting for PRS *or* AK (95% CI)	OR[a] adjusting for PRS *and* AK (95% CI)
SCC PRS	1.4 (1.31,1.49)	1.4 (1.31,1.50)
AK 365 days before any skin cancer diagnosis	0.93 (0.62,1.34)	0.89 (0.65,1.19)

Table B. Predictors for squamous cell carcinoma (SCC) in MGI.
[a] also adjusting for Age, gender, array, and PCs 1-4

PRS<br>(Number of Loci/SNPs)		Skin Cancer<br>n = 4,503<br>(1,501 / 3,002)[d]	Melanoma<br>n = 1,896<br>(617 / 1,279)[d]	Basal Cell Carcinoma<br>n = 1,303<br>(419 / 884)[d]	Squamous Cell Carcinoma<br>n = 836<br>(273 / 563)[d]
Melanoma specific<br>(14 / 16)<br>[mPRS-u]	PRS OR[a]<br>P-value[a]<br>AUC[b]<br>HL χ^2, P-value [c]<br>Brier Score	1.08 (1.05,1.12)<br>4.1×10^{-6}<br>0.52 (0.51,0.53)<br>3.3, 0.91<br>0.14	1.14 (1.09,1.2)<br>4.4×10^{-8}<br>0.54 (0.52,0.56)<br>4.5, 0.81<br>0.094	1.06 (1.01,1.13)<br>0.032<br>0.53 (0.51,0.55)<br>8.3, 0.41<br>0.093	1.03 (0.958,1.11)<br>0.43<br>0.52 (0.49,0.54)<br>12, 0.17<br>0.092
Basal cell carcinoma specific<br>(18 / 18)<br>[bPRS-u]	PRS OR[a]<br>P-value[a]<br>AUC[b]<br>HL χ^2, P-value [c]<br>Brier Score	1.09 (1.06,1.13)<br>1.5×10^{-7}<br>0.52 (0.51,0.53)<br>8.7, 0.37<br>0.14	1.05 (1,1.1)<br>0.04<br>0.52 (0.5,0.53)<br>24, 0.0027<br>0.094	1.28 (1.2,1.35)<br>2.3×10^{-16}<br>0.57 (0.55,0.59)<br>4.1, 0.85<br>0.093	1.06 (0.985,1.14)<br>0.12<br>0.52 (0.5,0.55)<br>14, 0.087<br>0.092
Squamous cell carcinoma specific<br>(2 / 2)<br>[sPRS-u]	PRS OR[a]<br>P-value[a]<br>AUC[b]<br>HL χ^2, P-value [c]<br>Brier Score	1.01 (0.978,1.04)<br>0.54<br>0.5 (0.49,0.51)<br>8.9, 0.35<br>0.14	0.991 (0.944,1.04)<br>0.72<br>0.51 (0.49,0.53)<br>11, 0.21<br>0.094	1.01 (0.952,1.07)<br>0.77<br>0.5 (0.48,0.52)<br>15, 0.069<br>0.093	1.02 (0.952,1.09)<br>0.54<br>0.51 (0.49,0.54)<br>14, 0.077<br>0.092
Shared PRS<br>(6 / 7)	PRS OR[a]<br>P-value[a]<br>AUC[b]<br>HL χ^2, P-value [c]<br>Brier Score	1.34 (1.3,1.39)<br>9.1×10^{-68}<br>0.58 (0.57,0.59)<br>8.3, 0.41<br>0.14	1.47 (1.4,1.54)<br>5.2×10^{-56}<br>0.6 (0.58,0.62)<br>11, 0.21<br>0.093	1.49 (1.41,1.58)<br>1.4×10^{-42}<br>0.61 (0.59,0.63)<br>10, 0.24<br>0.092	1.37 (1.28,1.48)<br>4.9×10^{-18}<br>0.59 (0.57,0.62)<br>11, 0.22<br>0.091
Skin Tanning Ability PRS<br>(20 / 26)	PRS OR[a]<br>P-value[a]<br>AUC[b]<br>HL χ^2, P-value [c]<br>Brier Score	1.39 (1.35,1.44)<br>2.7×10^{-84}<br>0.59 (0.58,0.6)<br>6.3, 0.62<br>0.14	1.51 (1.43,1.58)<br>1.4×10^{-61}<br>0.61 (0.59,0.62)<br>7.5, 0.48<br>0.093	1.54 (1.45,1.63)<br>4.3×10^{-48}<br>0.62 (0.6,0.64)<br>9, 0.34<br>0.092	1.51 (1.41,1.63)<br>8.2×10^{-29}<br>0.61 (0.59,0.64)<br>10, 0.26<br>0.09

Table C. Associations of Modified PRS with skin cancer traits in MGI**.** The subtype-specific PRS (mPRS-u, bPRS-u, and sPRS-u) consist of SNPs that were unique to that subtypes overall PRS (mPRS, bPRS, and sPRS). The shared skin cancer PRS represents the unweighted sum of risk allele counts of seven independent SNPs of six risk loci that are shared between the mPRS, bPRS, and the sPRS (**Figure 2**). The skin tanning ability PRS is based on 26 SNPs identified to be related to skin tanning ability in a previous GWAS meta-analysis study. The shared PRS and the skin saturation PRS have six overlapping SNPs.

[a] Association of each cancer with continuous PRS that were transformed to standard normal distribution. Point estimates, 95% confidence intervals and P- values are obtained by fitting Firth's Bias-Corrected Logistic Regression adjusted for age, sex, batch and PC1-4 to the full data.

[b] Area under the curve of the receiver operating characteristic (ROC) curve with 95% confidence intervals for the test data after fitting a model with the training data.

[c] Hosmer-Lemeshow Goodness-of-Fit Test for the test data after fitting a model with the training data.

[d] Number of cases in training / test set

PRS (Number of SNPs)		Skin cancer n = 4,503 (1,501 / 3,002)[d]	Melanoma n = 1,896 (617 / 1,279)[d]	Basal cell carcinoma n = 1,303 (419 / 884)[d]	Squamous cell carcinoma n = 836 (273 / 563)[d]
Skin cancer P < 5 x 10^{-9} (40)	PRS OR[a]	1.33 (1.28,1.37)	1.34 (1.28,1.41)	1.6 (1.51,1.69)	1.33 (1.24,1.42)
	P-value[a]	6.1x10^{-65}	2.4x10^{-35}	8.1x10^{-61}	1.7x10^{-15}
	AUC[b]	0.58 (0.57,0.59)	0.58 (0.56,0.6)	0.63 (0.61,0.65)	0.58 (0.55,0.61)
	HL χ^2, P-value [c]	9.3, 0.32	15, 0.064	15, 0.064	3.9, 0.86
	Brier Score	0.14	0.093	0.091	0.091
Skin cancer P < 5 x 10^{-8} (50)	PRS OR[a]	1.34 (1.3,1.39)	1.37 (1.3,1.43)	1.62 (1.53,1.72)	1.35 (1.26,1.45)
	P-value[a]	5.7x10^{-70}	5.3x10^{-39}	2.6x10^{-64}	1.9x10^{-17}
	AUC[b]	0.58 (0.57,0.59)	0.58 (0.56,0.6)	0.64 (0.62,0.66)	0.58 (0.56,0.61)
	HL χ^2, P-value [c]	9.1, 0.34	12, 0.16	16, 0.04	5.5, 0.71
	Brier Score	0.14	0.093	0.091	0.091
Skin cancer P < 5 x 10^{-7} (63)	PRS OR[a]	1.34 (1.3,1.39)	1.37 (1.31,1.43)	1.64 (1.55,1.74)	1.34 (1.25,1.44)
	P-value[a]	3.1x10^{-70}	6.1x10^{-39}	2.8x10^{-66}	2.6x10^{-16}
	AUC[b]	0.58 (0.57,0.59)	0.58 (0.57,0.6)	0.64 (0.62,0.66)	0.58 (0.55,0.61)
	HL χ^2, P-value [c]	3.6, 0.89	12, 0.16	11, 0.21	8, 0.43
	Brier Score	0.14	0.093	0.091	0.091
Skin cancer P < 5 x 10^{-6} (104)	PRS OR[a]	1.33 (1.29,1.38)	1.36 (1.3,1.43)	1.63 (1.54,1.73)	1.34 (1.25,1.44)
	P-value[a]	1x10^{-66}	7.9x10^{-37}	2.8x10^{-63}	5.4x10^{-16}
	AUC[b]	0.58 (0.57,0.59)	0.58 (0.57,0.6)	0.63 (0.61,0.65)	0.58 (0.56,0.61)
	HL χ^2, P-value [c]	3.1, 0.93	12, 0.13	7.8, 0.45	4.1, 0.85
	Brier Score	0.14	0.093	0.091	0.091
Skin cancer P < 5 x 10^{-5} (309)	PRS OR[a]	1.32 (1.28,1.37)	1.37 (1.31,1.44)	1.6 (1.51,1.7)	1.29 (1.2,1.38)
	P-value[a]	6.8x10^{-63}	3.6x10^{-39}	2.3x10^{-59}	3.2x10^{-12}
	AUC[b]	0.58 (0.57,0.59)	0.59 (0.58,0.61)	0.64 (0.62,0.66)	0.57 (0.55,0.6)
	HL χ^2, P-value [c]	5.8, 0.67	18, 0.025	15, 0.061	11, 0.21
	Brier Score	0.14	0.093	0.091	0.091
Skin cancer P < 5 x 10^{-4} (1473)	PRS OR[a]	1.21 (1.17,1.25)	1.27 (1.21,1.33)	1.4 (1.32,1.48)	1.17 (1.09,1.26)
	P-value[a]	1.9x10^{-30}	2.1x10^{-22}	1.8x10^{-29}	2.1x10^{-5}
	AUC[b]	0.56 (0.54,0.57)	0.57 (0.55,0.58)	0.59 (0.57,0.61)	0.55 (0.52,0.57)
	HL χ^2, P-value [c]	14, 0.071	9.4, 0.31	25, 0.0017	15, 0.061
	Brier Score	0.14	0.094	0.092	0.091
Melanoma P < 5 x 10^{-9} (6)	PRS OR[a]	1.21 (1.17,1.25)	1.32 (1.26,1.38)	1.2 (1.14,1.26)	1.19 (1.11,1.27)
	P-value[a]	1.3x10^{-31}	3.1x10^{-34}	9x10^{-11}	6.3x10^{-7}
	AUC[b]	0.55 (0.54,0.56)	0.58 (0.56,0.59)	0.55 (0.53,0.57)	0.56 (0.53,0.58)
	HL χ^2, P-value [c]	13, 0.11	9.8, 0.28	10, 0.26	24, 0.0024
	Brier Score	0.14	0.093	0.093	0.091
Melanoma P < 5 x 10^{-8} (9)	PRS OR[a]	1.26 (1.22,1.3)	1.4 (1.33,1.46)	1.27 (1.2,1.34)	1.22 (1.14,1.3)
	P-value[a]	4.2x10^{-44}	1.7x10^{-46}	8.2x10^{-17}	4x10^{-8}
	AUC[b]	0.56 (0.55,0.57)	0.59 (0.58,0.61)	0.57 (0.55,0.59)	0.56 (0.53,0.58)
	HL χ^2, P-value [c]	7, 0.53	6.5, 0.59	9.9, 0.27	15, 0.052
	Brier Score	0.14	0.093	0.093	0.091
Melanoma P < 5 x 10^{-7} (13)	PRS OR[a]	1.25 (1.21,1.29)	1.4 (1.34,1.47)	1.25 (1.18,1.32)	1.21 (1.13,1.3)
	P-value[a]	2.8x10^{-42}	6.5x10^{-47}	1.2x10^{-14}	1.5x10^{-7}
	AUC[b]	0.56 (0.55,0.58)	0.59 (0.58,0.61)	0.56 (0.54,0.58)	0.56 (0.53,0.58)
	HL χ^2, P-value [c]	6.5, 0.59	3.9, 0.86	11, 0.22	10, 0.25
	Brier Score	0.14	0.093	0.093	0.091
Melanoma P < 5 x 10^{-6} (27)	PRS OR[a]	1.22 (1.18,1.26)	1.35 (1.29,1.41)	1.21 (1.15,1.28)	1.2 (1.12,1.29)
	P-value[a]	1.1x10^{-32}	3.6x10^{-36}	3.2x10^{-11}	5.2x10^{-7}
	AUC[b]	0.56 (0.55,0.57)	0.58 (0.56,0.6)	0.55 (0.53,0.57)	0.55 (0.53,0.58)
	HL χ^2, P-value [c]	15, 0.054	7.6, 0.47	18, 0.022	14, 0.076
	Brier Score	0.14	0.093	0.093	0.091
Melanoma P < 5 x 10^{-5} (156)	PRS OR[a]	1.15 (1.11,1.19)	1.24 (1.19,1.3)	1.16 (1.1,1.23)	1.15 (1.07,1.24)
	P-value[a]	5.8x10^{-17}	5.8x10^{-20}	1.4x10^{-7}	1x10^{-4}
	AUC[b]	0.54 (0.53,0.55)	0.56 (0.54,0.58)	0.53 (0.51,0.55)	0.54 (0.51,0.56)
	HL χ^2, P-value [c]	9.2, 0.32	7.4, 0.49	25, 0.0017	14, 0.088
	Brier Score	0.14	0.094	0.093	0.092
Melanoma P < 5 x 10^{-4} (1193)	PRS OR[a]	1.07 (1.03,1.1)	1.1 (1.05,1.15)	1.11 (1.05,1.17)	1.07 (0.996,1.15)
	P-value[a]	7.2x10^{-5}	0.00011	0.00046	0.066
	AUC[b]	0.52 (0.51,0.53)	0.54 (0.52,0.55)	0.52 (0.5,0.54)	0.51 (0.48,0.53)
	HL χ^2, P-value [c]	3.8, 0.88	20, 0.011	13, 0.12	21, 0.0075
	Brier Score	0.14	0.094	0.093	0.092

Table D. Association of PRS based on UKB summary statistics with skin cancer traits in MGI using various P-value thresholds

[a] Association of each cancer with continuous PRS that were transformed to standard normal distribution. Point estimates, 95% confidence intervals and P- values are obtained by fitting Firth's Bias-Corrected Logistic Regression adjusted for age, sex, batch and PC1-4 to the full data.

[b] Area under the curve of the receiver operating characteristic (ROC) curve with 95% confidence intervals for the test data after fitting a model with the training data.

[c] Hosmer-Lemeshow Goodness-of-Fit Test for the test data after fitting a model with the training data

[d] Number of cases in training / test set

PRS; Proportion of causal variants		Skin cancer n = 4,503 (1,501 / 3,002)[d]	Melanoma n = 1,896 (617 / 1,279)[d]	Basal cell carcinoma n = 1,303 (419 / 884)[d]	Squamous cell carcinoma n = 836 (273 / 563)[d]
Skin cancer 100%	PRS OR, P-value[a]	1.2 (1.16,1.24), 7x10^{-27}	1.22 (1.16,1.28), 2.3x10^{-16}	1.31 (1.24,1.39), 2.7x10^{-20}	1.17 (1.08,1.25), 3.1x10^{-5}
	AUC[b]	0.55 (0.54,0.56)	0.56 (0.54,0.58)	0.57 (0.55,0.59)	0.54 (0.51,0.56)
	HL χ^2, P-value[c]	4.8, 0.78	7.2, 0.51	18, 0.019	4.1, 0.84
	Brier Score	0.14	0.094	0.093	0.091
	Nagelkerke R^2	0.0083	0.0057	0.015	0.0056
Skin cancer 10%	PRS OR, P-value[a]	1.21 (1.17,1.25), 8.2x10^{-30}	1.24 (1.18,1.3), 4.9x10^{-18}	1.33 (1.26,1.41), 1.2x10^{-22}	1.18 (1.1,1.27), 6.7x10^{-6}
	AUC[b]	0.55 (0.54,0.56)	0.56 (0.55,0.58)	0.58 (0.56,0.6)	0.54 (0.52,0.57)
	HL χ^2, P-value[c]	4.4, 0.82	5.8, 0.67	15, 0.061	8, 0.43
	Brier Score	0.14	0.094	0.093	0.091
	Nagelkerke R^2	0.0092	0.0064	0.017	0.0061
Skin cancer 1%	PRS OR, P-value[a]	1.3 (1.26,1.35), 3x10^{-55}	1.33 (1.27,1.4), 7.2x10^{-32}	1.51 (1.42,1.6), 3.8x10^{-44}	1.28 (1.19,1.38), 1.4x10^{-11}
	AUC[b]	0.57 (0.56,0.58)	0.58 (0.56,0.6)	0.61 (0.59,0.63)	0.57 (0.54,0.59)
	HL χ^2, P-value[c]	8.6, 0.37	5.6, 0.69	8, 0.43	14, 0.076
	Brier Score	0.14	0.093	0.092	0.091
	Nagelkerke R^2	0.017	0.013	0.033	0.012
Skin cancer 0.1%	PRS OR, P-value[a]	1.03 (0.998,1.07), 0.062	1.02 (0.977,1.08), 0.32	1.07 (1.01,1.13), 0.017	1.04 (0.972,1.12), 0.23
	AUC[b]	0.5 (0.49,0.51)	0.5 (0.49,0.52)	0.52 (0.5,0.54)	0.5 (0.47,0.52)
	HL χ^2, P-value[c]	14, 0.079	4.4, 0.82	11, 0.23	9.1, 0.33
	Brier Score	0.14	0.094	0.093	0.092
	Nagelkerke R^2	0.0012	0.00014	0.00062	0.0016
Skin cancer 0.01%	PRS OR, P-value[a]	1.06 (1.03,1.1), 0.00017	1.03 (0.978,1.08), 0.29	1.1 (1.04,1.16), 0.0016	1.11 (1.03,1.19), 0.004
	AUC[b]	0.51 (0.5,0.52)	0.49 (0.48,0.51)	0.53 (0.51,0.55)	0.54 (0.51,0.56)
	HL χ^2, P-value[c]	21, 0.0064	12, 0.17	7.6, 0.47	9.7, 0.29
	Brier Score	0.14	0.094	0.093	0.092
	Nagelkerke R^2	0.0017	8.9e-06	0.0011	0.00047
Skin cancer 0.001%	PRS OR, P-value[a]	1.08 (1.04,1.11), 1x10^{-5}	1.05 (1,1.1), 0.041	1.1 (1.04,1.17), 0.001	1.08 (1,1.16), 0.04
	AUC[b]	0.51 (0.5,0.52)	0.52 (0.5,0.53)	0.52 (0.5,0.54)	0.52 (0.49,0.54)
	HL χ^2, P-value[c]	15, 0.054	11, 0.21	11, 0.19	5.7, 0.69
	Brier Score	0.14	0.094	0.093	0.092
	Nagelkerke R^2	0.003	1e-04	0.0014	0.001
Melanoma 100%	PRS OR, P-value[a]	1.08 (1.05,1.12), 2.6x10^{-6}	1.14 (1.09,1.2), 6.8x10^{-8}	1.07 (1.01,1.14), 0.018	1.01 (0.935,1.08), 0.89
	AUC[b]	0.52 (0.51,0.53)	0.54 (0.53,0.56)	0.53 (0.51,0.55)	0.51 (0.49,0.54)
	HL χ^2, P-value[c]	6.5, 0.59	8.6, 0.38	18, 0.024	18, 0.023
	Brier Score	0.14	0.094	0.093	0.092
	Nagelkerke R^2	0.0024	0.0016	0.00018	0.00072
Melanoma 10%	PRS OR, P-value[a]	1.08 (1.05,1.12), 2x10^{-6}	1.14 (1.09,1.2), 4.8x10^{-8}	1.07 (1.01,1.14), 0.016	1.01 (0.936,1.08), 0.87
	AUC[b]	0.52 (0.51,0.53)	0.54 (0.53,0.56)	0.53 (0.51,0.55)	0.51 (0.49,0.54)
	HL χ^2, P-value[c]	5.7, 0.68	9.2, 0.32	18, 0.023	18, 0.024
	Brier Score	0.14	0.094	0.093	0.092
	Nagelkerke R^2	0.0024	0.0017	0.00019	0.00071
Melanoma 1%	PRS OR, P-value[a]	1.1 (1.06,1.13), 4.4x10^{-8}	1.16 (1.11,1.22), 4.6x10^{-10}	1.09 (1.03,1.15), 0.0047	1.02 (0.948,1.1), 0.61
	AUC[b]	0.53 (0.51,0.54)	0.55 (0.53,0.57)	0.53 (0.51,0.55)	0.52 (0.49,0.54)
	HL χ^2, P-value[c]	6.8, 0.56	11, 0.22	21, 0.0066	14, 0.09
	Brier Score	0.14	0.094	0.093	0.092
	Nagelkerke R^2	0.003	0.0024	0.00034	0.00054
Melanoma 0.1%	PRS OR, P-value[a]	1.19 (1.16,1.23), 2.2x10^{-26}	1.32 (1.26,1.38), 1.2x10^{-30}	1.19 (1.12,1.26), 2.5x10^{-9}	1.14 (1.06,1.22), 0.00048
	AUC[b]	0.55 (0.54,0.56)	0.58 (0.57,0.6)	0.55 (0.53,0.57)	0.55 (0.52,0.57)
	HL χ^2, P-value[c]	22, 0.0055	22, 0.0047	21, 0.0078	20, 0.0097
	Brier Score	0.14	0.093	0.093	0.092
	Nagelkerke R^2	0.0085	0.011	0.0042	0.00015
Melanoma 0.01%	PRS OR, P-value[a]	1.27 (1.23,1.31), 7.6x10^{-46}	1.39 (1.33,1.46), 2.8x10^{-44}	1.29 (1.22,1.37), 7.4x10^{-19}	1.26 (1.17,1.35), 3.5x10^{-10}
	AUC[b]	0.57 (0.56,0.58)	0.6 (0.58,0.61)	0.57 (0.55,0.59)	0.56 (0.54,0.59)
	HL χ^2, P-value[c]	14, 0.092	11, 0.19	26, 0.00091	6.3, 0.61
	Brier Score	0.14	0.093	0.093	0.091
	Nagelkerke R^2	0.012	0.018	0.016	0.0087
Melanoma 0.001%	PRS OR, P-value[a]	1.28 (1.24,1.32), 7.7x10^{-50}	1.42 (1.36,1.49), 3.2x10^{-50}	1.29 (1.22,1.37), 5.1x10^{-19}	1.27 (1.19,1.37), 1.3x10^{-11}
	AUC[b]	0.57 (0.56,0.58)	0.6 (0.58,0.62)	0.57 (0.55,0.59)	0.57 (0.54,0.59)
	HL χ^2, P-value[c]	12, 0.13	7.5, 0.48	14, 0.083	11, 0.18
	Brier Score	0.14	0.093	0.093	0.091
	Nagelkerke R^2	0.015	0.024	0.016	0.0087

Table E. Association of PRS based on UKB summary statistics with skin cancer traits in MGI using LDpred with various assumed proportions of causal variants

Footnotes:

[a] Association of each cancer with continuous PRS that were transformed to standard normal distribution. Point estimates, 95% confidence intervals are obtained by fitting Firth's Bias-Corrected Logistic Regression adjusted for age, sex, batch and PC1-4 to the full data.

[b] Area under the curve of the receiver operating characteristic (ROC) curve with 95% confidence intervals for the test data after fitting a model with the training data.

[c] Hosmer-Lemeshow Goodness-of-Fit Test for the test data after fitting a model with the training data.

[d] Number of cases in training / test set
